# Measuring population health using health expectancy estimates from morbidity and mortality databases

**DOI:** 10.1371/journal.pone.0302174

**Published:** 2024-05-21

**Authors:** Marc Carreras, Pere Ibern, José María Inoriza

**Affiliations:** 1 Serra Hunter Fellow, University of Girona, Girona, Spain; 2 Research Group on Health Services and Health Outcomes (GRESSIRES), Palamós, Spain; 3 Centre for Research in Health and Economics (CRES), Pompeu Fabra University, Barcelona, Spain; 4 Fundació Hospital de Palamós—Serveis de Salut Integrats Baix Empordà (SSIBE), Palamós, Spain; Victoria University, AUSTRALIA

## Abstract

The progressive incorporation of quality of life indicators in health planning meets a critical need: The evaluation of the performance of health services, which are under stress by multiple causes, but in particular by an ageing population. In general, national health plans rely on health expectancies obtained using the Sullivan method. The Sullivan health expectancy index combines age-specific mortality rates and age-specific prevalence of healthy life, obtained from health surveys. The objective of this work is to investigate an equivalent estimation, using available information from morbidity and mortality datasets. Mortality and morbidity information, corresponding to years 2016 and 2017, was obtained for the population of the county of Baix Empordà (Catalonia), N = 91,130. Anonymized individual information on diagnoses, procedures and pharmacy consumption contained in the individual clinical record (ICD and ATC codes), were classified into health states. Based on the observed health transitions and mortality, life expectancies by health state were obtained from a multistate microsimulation model. Healthy life expectancies at birth and 65 years for females and males were respectively HLE_0_^female^ = 39.94, HLE_0_^male^ = 42.87, HLE_65_^female^ = 2.43, HLE_65_^male^ = 2.17. These results differed considerably from the Sullivan equivalents, e.g., 8.25 years less for HLE_65_^female^, 9.26 less for HLE_65_^male^. Point estimates for global life expectancies at birth and 65 years of age: LE_0_^female^ = 85.82, LE_0_^male^ = 80.58, LE_65_^female^ = 22.31, LE_65_^male^ = 18.86. Health indicators can be efficiently obtained from multistate models based on mortality and morbidity information, without the use of health surveys. This alternative method could be used for monitoring populations in the context of health planning. Life Expectancy results were consistent with the standard government reports. Due to the different approximation to the concept of health (data-based versus self-perception), healthy life expectancies obtained from multistate micro simulation are consistently lower than those calculated with the standard Sullivan method.

## Introduction

The progressive incorporation of quality of life indicators in health planning meets a critical need: The evaluation of the performance of health services, which are under stress by multiple causes, but in particular by an ageing population. There is a growing interest in measuring not only the number of years lived, but also the quality, adding concepts such as chronic disease burden or self-perceived health status. According to its most extended definition, Life Expectancy (LE) can be obtained as the average future lifetime for a person of a certain specific age, assuming that current age-specific mortality rates remain constant [1]. However, despite the broad consensus on the calculation of LE, the measurement of quality of life remains a more complex issue [2–7]

Different types of Health Expectancies (HE), are obtained as mixed indicators, combining LE and a certain approach to the concept of health. For example, Healthy Life Expectancy (HLE) can be defined as the average number of years a hypothetical cohort would life in good health, subject to the current mortality and morbidity conditions [8]. In general, health expectations are usually calculated based on the Sullivan method, i.e. by combining a given prevalence rate and the age-specific mortality rate [9–11]. This method is the standard for comparison between different populations and also for comparison over time for the same population [12].

The critical element in the calculation of HE is the measurement of the concept of health, which can be approached from different dimensions, such as the individual self-perceived health, occurrence of chronic diseases or disability. For example, HLE uses the subjective perception of health obtained from population surveys [10, 13, 14]. The measure of self-perceived health is inferred from one or more simple questions, in which the respondent answers according to his own or her assessment. There is an European agreement on the use of common questions for the purpose of standardisation [14].

Despite the consensus on the definition of indicators such as the HLE, it is important to consider its dependence on the availability of health surveys, usually conducted only on a limited basis, with relatively small but representative population samples, for certain periods and population areas, and implying considerable economic costs. For this reason, an important question that emerges is whether the enormous amount of clinical-administrative information stored by health systems can be used to obtain equivalent HE indicators. An affirmative answer to the question would imply that HE can be produced more efficiently.

This idea is based upon two considerations. First, demographic and clinical information can be easily transformed into categories equivalent to health states [15–18], i.e. adjustment or classification systems for the clinical management of patients. For example, 3M™ Clinical Risk Groups (CRG) software approximates the health status of individuals in a population, described from a clinical point of view (chronic disease burden), from the set of diagnostic and consumption codes of pharmacies registered during a given period [15]. Second, individual lifetime trajectories and global expectations can be obtained by integrating demographic, clinical and mortality information into micro simulation models [19–22].

The development of new efficient HLE methodologies is important because HLE is considered an essential indicator of the overall performance of any healthcare system. The previous affirmation is evidenced by the fact that HLE indicators are well established in the EU statistics and are present in most European countries’ health plans.

However, without questioning the suitability of the Sullivan method for international comparisons between countries, a research question that arises is whether an efficient alternative to standard HLE indicators can be obtained. We refer, for example, to relatively small populations, sometimes located in well-defined geographical areas, sharing a common health plan, clinical data, and financing scheme. In these situations, LE and HLE can be used as synthetic performance measures of the outcomes of healthcare services [23]. In line with the above ideas, the main objective of this study is to obtain an alternative estimation of HLE inferred from demographic, morbidity, and mortality data available for a specific population. Throughout this work, and just for comparative purposes, we show the HLE calculation using the standard Sullivan’s estimate [24], conducted by the Catalan government for the same population. Methodological and conceptual differences related to HLE constructions (data versus self-perception based) will be discussed in the further sections of the article.

## Material and methods

### Population data

Individual data of the population of the county of Baix Empordà (Catalonia) was collected ex-post for the consecutive years 2016 and 2017. The resident population, covered by the public insurance scheme, was composed of 91,130 individuals, of whom we had complete follow-up in the health system of N = 87,850 (individuals included in the study). The data collection was conducted in the first quarter of 2019. The set of anonymized individual information included demographic, morbidity, and mortality data. The particular subset of morbidity data included exhaustive individual information on diagnoses, procedures and pharmacy consumption contained in the individual clinical record, according to the International Classification of Diseases (ICD) and Anatomical Therapeutic Chemical Classification system (ATC) codes [25, 26].

The dataset was made possible thanks to the support of Serveis de Salut Integrats Baix Empordà (SSIBE, www.ssibe.cat), an integrated healthcare management organisation responsible for the integrated public provision of health services for the population living in the county of Baix Empordà (Catalonia). The services provided included acute inpatient care, acute outpatient care, primary care, pharmacy prescriptions, diagnostic tests, emergencies, and long-term residential care services. The framework of organization and delivery of healthcare services described above is equivalent to an integrated care delivery system.

For clinical management purposes the Baix Empordà population was individually classified into health states according to the 3M™ Clinical Risk Groups (CRG) software (version 1.9). Individuals were classified into mutually exclusive categories according to their clinical data and demographic characteristics. The original CRG health status classification aggregates individuals into nine categories: 1. Healthy; 2. History of significant acute disease; 3. Single minor chronic disease; 4. Minor chronic disease in multiple organ systems; 5. Single dominant or moderate chronic disease; 6. Significant chronic disease in multiple organ systems; 7. Dominant chronic disease in three or more organ systems; 8. Dominant and metastatic malignancies and 9. Catastrophic conditions.

Throughout this article, we used the CRG classification as a proxy of the health status of the population. However, with the objective of minimizing the number of groups with a reduced number of individuals, we aggregated individual morbidity histories into six health status: 1. Healthy; 2. Significant acute disease; 3. Minor chronic disease; 4. Significant chronic disease in one or two organ systems; 5. Significant chronic disease in three or more organ systems—Catastrophic conditions; 6. Dominant and metastatic malignancies.

ICD codes on diagnostics and procedures and ATC drug codes related to the 87,850 individuals included in the study were collected between January 1, 2016, and December 31, 2017. A number of 758 deaths corresponding to individuals in the study population were reported during the year 2017.

**Fig 1:**
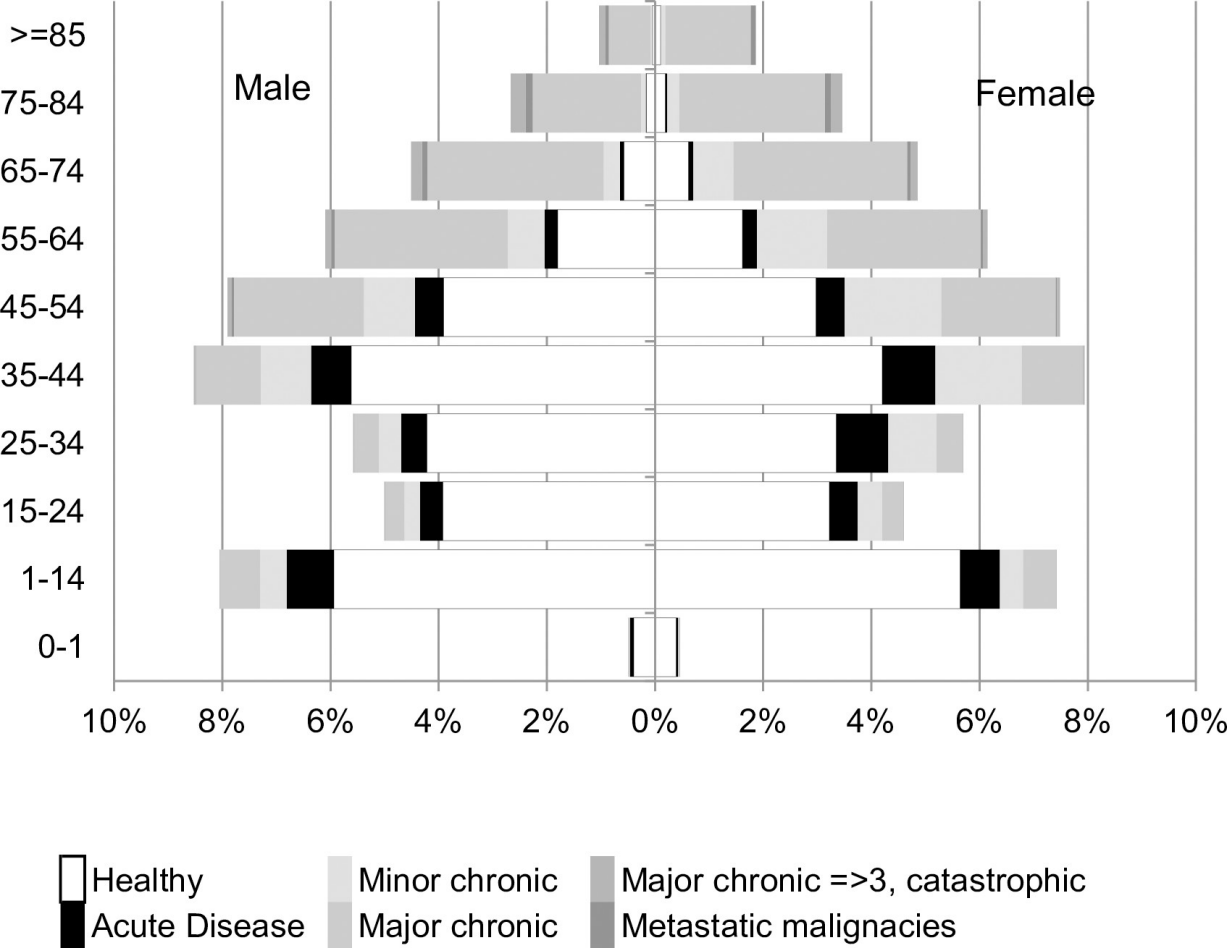
Study population at December 31, 2016 (N = 87,850).

The demographic characteristics and the CRG status of the population at December 31, 2016 are shown in Fig 1 and S1 Table.

**Fig 2:**
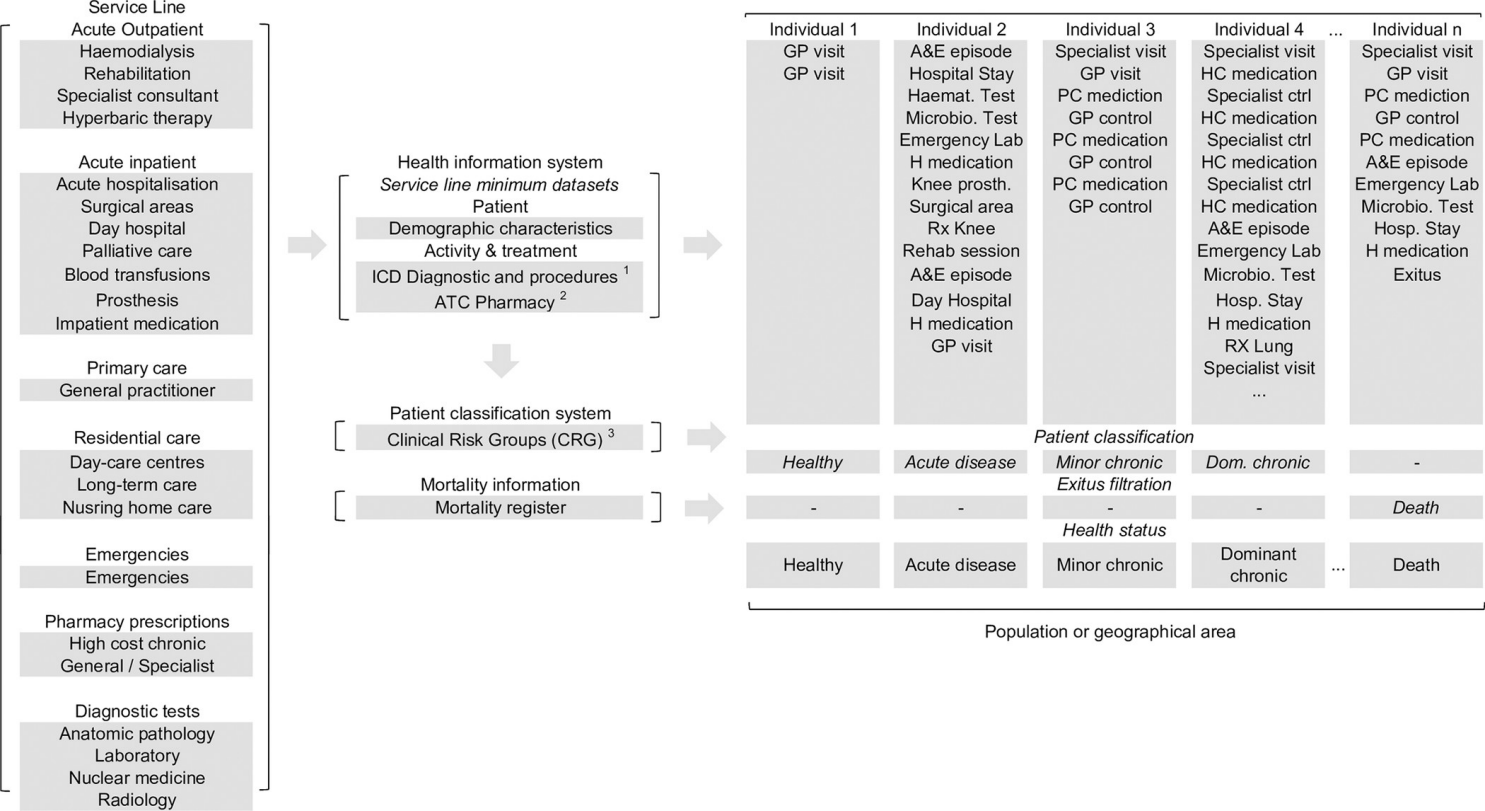
Transformation of individual morbidity and mortality data to health status. 1. International Statistical Classification of Diseases and Related Health Problems (ICD); 2. Anatomical Therapeutic Chemical (ATC) Classification; 3. Clinical Risk Groups (CRGs).

Fig 2 shows how the individual data (individual ICD and ATC codes) [25, 26], coming from different clinical sources, were transformed into CRG categories equivalent to health status [15].

### Healthy Life Expectancy by multistate micro simulation (HLE-MMS)

Based on the individual health status transitions observed between 2016 and 2017, life expectancies by health state, were obtained from a multistate Markov chain microsimulation model.

According to the fundamental Markov chain assumptions, time in the process takes discrete values *t = 1*,*2*, *…*, *n* (natural years), as well as the space of states *X*_*t*,*1*_,*X*_*t*,*2*_,*…*,*X*_*t*,*m*_. Consequently, the health status of an individual for a specific year was assumed depending only on the health status observed in the previous year. In other words, the last observed status summarised the health history of the individual [18–21].


$$P(X_{t}=x_{t}|X_{t-1}=x_{t-1},\ldots ,X_{2}=x_{2},X_{1}=x_{1})=P(X_{t}=x_{t}|X_{t-1}=x_{t-1})$$ (1)

The transition probabilities required in the model were assumed stationary, i.e., constant over time and were estimated from the individual health status transitions observed for the couple of years 2016–2017. Following the previous definitions, we considered six health states plus death as a final absorbing state at the second year (2017). Moreover, we considered sex and ten age groups: < 1 year of age, 1–14 years of age, 15–24, 25–34, 35–44, 45–54, 55–64, 65–74, 75–84,> = 85. The design of the age groups balanced two aspects: first, homogeneity of morbidity and mortality within the groups and second, ensure a minimum number of individuals in each cell [sex * age-group * health-state].

**Fig 3:**
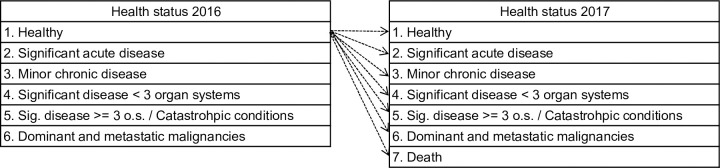
Health status transition model. "Sig.": significant; "o.s.": organ systems.

The health transition model is described in Fig 3.

The maximum likelihood estimator of the stationary transition probabilities was simply obtained as counts or frequencies:

$${\hat{p}}_{\mathrm{ij}}(age,t)=s_{\mathrm{ij}}(age,t)/s_{i}(age,t-1)$$ (2)

where *t = 1*, *2*,…, *T* are the times of observation, i, *j = 1*, *2*, *…*, *m* are the states of the process and S_*ij*_ are the number of individuals having state *j* at time *t*, having state *i* at time *t-1*.

The result was an initial set of 20 transition matrices, corresponding to the defined age groups, 10 for men and 10 for women (S2 Table). According to the design of the experiment, these probabilities were age-group centred. Then, except for the first age group (< 1 year of age), we interpolated the yearly transition probabilities using cubic splines. The result was a set of 182 matrices describing the yearly transitions, 91 matrices for men and 91 for women (0 to > = 90 years of age) (S3 Table). This design (first estimate transition probabilities by age group and then interpolate annual probabilities) ensures a minimum number of individuals in each cell [sex * age-group * health-state] and guarantees a robust estimation.

In the next step, we generated a sample of random lifetime trajectories according to the observed transition probabilities, N_1_ = 10,000 females, and N_2_ = 10,000 males. The algorithm started assigning an initial health status for a standard individual. Such initial assignation reproduced the composition of the population group < 1 year of age. Hereinafter, for each individual, the model generated a sequence of cycles from birth until death (final absorbing state). A new cycle in the simulation represented an additional year of life. According to the Monte Carlo simulation scheme, changes in the health status were obtained generating pseudo-random numbers and comparing them to the transition probabilities. The final result was a random sample from which we can calculate Health Expectancies according to the standard demographic formula:

$$e_{x,j}=\frac{\sum_{y\geq x}L_{y,j}}{l_{x}}\;j=1,2,\ldots k$$ (3)

*j = 1*, *2*, *…*, *7* were the defined states of the process, *L*_*y*,*j*_ the number of person years lived at age *y* in the state *j* and *l*_x_ the number of survivors at age *X*.

The healthy life expectancy metrics from this model (HLE-MMS) corresponds to the CRG status “1. Healthy”.

Graphics, data management and statistical calculations were conducted using the Microsoft Office Suite and the Office Visual Basic for Applications (VBA) programming libraries [27].

### Healthy Life Expectancy by Sullivan’s method (HLE-Sullivan)

Considering the county of Baix Empordà as a specific geographical area in Catalonia, HLE estimates of the study population can be obtained from government official reports. Within the period of study, the Catalan Health Department (Departament de Salut) calculated global HLE estimates, for the general Catalan population. HLE estimates were periodically calculated by sex, at different ages of interest, using mortality and self-perception of health information. Mortality data was systematically obtained from the Catalan Register of Mortality (RMC) and the information on self-perception of health from the Catalan Health Survey (ESCA) [13, 24]. Moreover, the self-perception of health question in the survey included the standard answers: 1. Very good (VGOOD); 2. Good (GOOD); 3. Fair (FAIR); 4. Bad (BAD); 5. Very bad (VBAD); according to the European Health Interview Survey (EHIS) and the definition of dimensions of the EU statistics on income and living conditions (EU-SILC) methodology [14]. Since, question and answers were exactly the same, the HLE indicators obtained from the Catalan government health department are comparable to the equivalent European standards from the European Health and Life Expectancy Information System (EHLEIS) [12, 14].

Throughout the study we, and just for comparative purposes, used the global Catalan HLE estimates calculated for 2017 as a HLE proxy for the Baix Empordà population for the period 2016–2017.

### Ethical considerations

The study was conducted in compliance with the Declaration of Helsinki (version in force; approved at the 64th General Assembly, Fortaleza, Brazil, October 2013) and in accordance with Law 14/2007, of July 3, on Biomedical Research.

The processing of personal and health data was in accordance with Regulation (EU) 2016/679 of the European Parliament and of the Council of 27 April 2016 on the protection of natural persons with regard to the processing of personal data and on the free movement of such data and repealing Directive 95/46/EC (GDPR) and in the Organic Law 3/2018, of 5 December, on the protection of personal data and guarantee of digital rights (LOPDGDDD), published in the BOE on 6 December 2018.

Only information related to the study was recorded. The database was pseudo-anonymized by a member of the SSIBE Research Department, ensuring technical and functional separation with the research team.

It was considered unnecessary to obtain informed consent for access to the patients’ EHRs based on the following considerations:

- This was a retrospective study.
- The data extraction and recording system designed allowed for technical and functional separation.
- No specific recruitment activity was performed, and all data were displayed in an aggregated format that does not allow the identification of specific patients.

## Results

### HLE-MMS: Combining mortality and available data on individual morbidity

The output of the multistate micro simulation model is a random sample of individual lifetime trajectories. A numerical approach of the global Life Expectancy function can be obtained from the sample just applying the standard demographic formulas. Moreover, the individual health status changes, along the lifespan, were recorded in the simulation data. Consequently, specific LE functions were also split according to the health status defined in the model: 1. Healthy; 2. Significant acute disease; 3. Minor chronic disease; 4. Significant chronic disease in one or two organ systems; 5. Significant chronic disease in three or more organ systems—Catastrophic conditions; 6. Dominant and metastatic malignancies. We assumed that the LE function for the health status ‘1. Healthy’ is equivalent to the HLE indicator obtained from the for the multistate micro simulation model (HLE-MMS).

**Fig 4:**
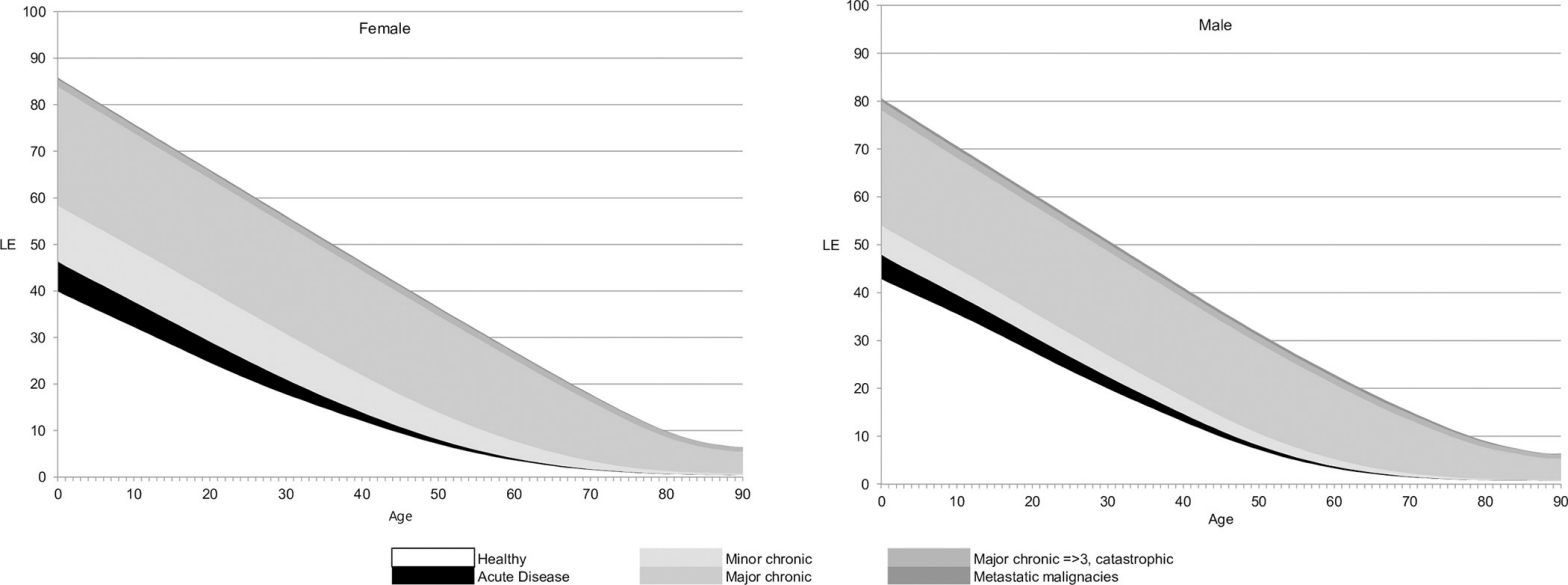
Health Expectancies.

Full numerical approximations of the LE function by health status are shown in the Fig 4. HLE-MMS point estimates at birth and 65 years for females and males were respectively HLE-MMS_0_^female^ = 39.94; HLE-MMS_0_^male^ = 42.87; HLE-MMS_65_^female^ = 2.43; HLE-MMS_65_^male^ = 2.17.

Finally, full Life Expectancies can also be obtained from the HLE-MMS model, considering all the coloured area in the Fig 4 (defined as LE-MMS). Point estimates at birth and 65 years of age: LE-MMS_0_^female^ = 85.82; LE-MMS_0_^male^ = 80.58; LE-MMS_65_^female^ = 22.31; LE-MMS_65_^male^ = 18.86.

### HLE-Sullivan: Combining mortality and standard health survey information

Since specific indicators of HLE-Sullivan for the population of the area of Baix Empordà were not available, in accordance with the methodology, we used the Catalan government estimates on mortality, demographic and health indicators, published for the year 2017. According to the official Catalan report [24] the values of HLE values are shown in the Table 1. HLE-Sullivan (HLE-S) point estimates at birth and 65 years for females and males were respectively HLE-S_0_^female^ = 66.08; HLE-S_0_^male^ = 66.62; HLE-S_65_^female^ = 10.68; HLE-S_65_^male^ = 11.43.

**Table 1:** HLE-S. Catalonia 2017.

	Female		Male	
Age (years)	HLE-S	SE	HLE-S	SE
0	66.08	0.6539	66.62	0.5869
1	65.23	0.6554	65.78	0.5883
5	61.5	0.6496	62.12	0.5805
10	56.8	0.6443	57.26	0.5781
15	51.86	0.6429	52.51	0.5711
20	47.24	0.6317	47.68	0.5681
25	42.35	0.6292	42.96	0.5607
30	37.57	0.6239	38.35	0.5519
35	33.18	0.6113	33.64	0.5476
40	28.96	0.5973	29.32	0.5358
45	24.87	0.5839	24.95	0.5285
50	20.82	0.5681	20.72	0.5184
55	17.1	0.551	17.28	0.4984
60	14.01	0.5271	14.17	0.4797
65	10.68	0.5027	11.43	0.4579
70	7.81	0.4716	8.96	0.4306
75	5.78	0.4178	6.73	0.3963
80	4.05	0.3794	4.15	0.3603

SE Standard errors.

Source: Freitas-Ramírez A, Puigdefàbregas-Serra A, Ribas G, Molina P [24].

As can be appreciated, the values obtained for the HLE-MMS indicators differed considerably from those obtained in the Table 1 for the HLE-Sullivan equivalent, e.g., 8.25 years less for HLE_65_^female^, 9.26 less for HLE_65_^male^.

### Life Expectancy

Given that the two versions of the HLE indicators were considerably different, an interesting exercise consists in comparing full life expectancies obtained from the multistate micro simulation with those obtained from the standard mortality model. For that purpose, we compared the LE obtained from the HLE-MMS model with the official standard reports on mortality and LE for Catalonia in 2017. We considered two sources of government data: 1. Catalan government reports [24] and 2. Spanish national institute of statistics (INE) reports [28], see Table 2.

**Table 2:** Life Expectancy, LE-MMS vs Government reports. Catalonia 2017.

	SSIBE—2017^1^			Catalonia—2017			Spain—2017		
Age	Female	Male	Total	Female	Male	Total	Female	Male	Total
0	85.82	80.58	83.20	86.16	80.71	83.5	85.73	80.37	83.09
10	75.86	70.60	73.23	76.41	70.97	73.75	75.99	70.68	73.37
20	65.99	60.74	63.36	66.47	61.08	63.84	66.06	60.77	63.46
30	56.09	50.91	53.50	56.57	51.27	53.99	56.17	50.99	53.62
40	46.23	41.05	43.64	46.72	41.54	44.2	46.33	41.26	43.84
50	36.49	31.52	34.02	37.05	32.03	34.62	36.69	31.82	34.31
60	26.99	22.86	24.97	27.79	23.25	25.62	27.44	23.13	25.37
70	17.82	15.20	16.60	18.87	15.45	17.31	18.59	15.37	17.11
80	9.86	8.98	9.49	10.71	8.7	9.89	10.56	8.72	9.8
90	6.45	6.42	6.44	4.91	4.14	4.67	4.86	4.24	4.67

1. Global life expectancies obtained from the HLE-MMS model.

Source: Freitas-Ramírez A, Puigdefàbregas-Serra A, Ribas G, Molina P [24]; INE, 2021. Esperanza de vida a diferentes edades [28].

Although HLE values based on individual morbidity data substantially differed from the official estimates based on self-reported health surveys (previous sections), the global LE indicators from both approaches were very close. According to the Catalan government data, the point estimates for LE at birth were: LE_0_^female^ = 86.16; LE_0_^male^ = 80.71. According to the INE data for Catalonia, the point estimates for LE at birth were: LE_0_^female^ = 85.73; LE_0_^male^ = 80.37. This similarity is maintained along all the range of ages except for the elder groups (> = 90 years of age). For these groups, the HLE-MMS model tend to overestimate LE in approximately 1.77 years.

Therefore, setting aside the different conception of HE, and apart from eldest individuals, the proposed approach converges on the same values for the LE estimates.

## Discussion

A first aspect to discuss is the different concept of health embedded in the two approaches compared in the article. The significant difference in the results is simply a reflection of such different conception of health status. Beyond the 1947 World Health Organization’s (WHO) definition of health “… complete physical, mental and social wellbeing…”, the literature reports difficulties in establishing a common and uniform concept of health. More recent approaches consider healthcare organisations, health care workers and patients as different agents with a non-uniform perspective on health [29]. Moreover, focusing on the patient context, the self-perception of health is a psychological and cultural convention susceptible to significant variability among individuals [30]. Other authors conclude that the inference of health status from clinical records on attended morbidity approximates to the subjective perception of health and vice versa. However, certain socio-demographic factors modulate the individual perception. Different studies described only moderate or fair agreement comparing self-reported morbidity and pharmacy prescription. Individual factors such as age, gender, marital status, education, poor-delayed recall, depression and polypharmacy were significantly associated with discordance between morbidity measures [31–33].

The research team considered to calculate the specific HLE-Sullivan estimate for the study population (instead of the global Catalan estimate), since it was feasible from the data available. Although, it would result in a more precise HLE estimate, since the focus of the study is the alternative multistate micro simulation method, the choice of the team was not to go further on the Sullivan estimates.

Methodologically, the proposed model can be classified into the *microsimulation of healthy life* type, described in detail in the literature [34, 35]. Moreover, the fundamental steps of the process, such as the estimation of parameters or the sampling method, are described in the aforementioned literature. Such microsimulation typology retains the advantages of models based on incidence, but without the dependence on rarely available, longitudinal surveys. These advantages mainly include obtaining an expected (mean) value from the set of simulated trajectories and the variability around that value. In respect to the existing research, the novelty of the proposed model is the use of clinically interpretable health states that can be directly obtained from clinical databases.

According to the literature, mortality statistics are fundamental to public decision making. Mortality is highly variable depending on time and location and is subject to well-known biases, which are particularly relevant in pandemic contexts, such as COVID-19 [36]. In these contexts, using the proposed method, countries or subnational locations that have reliable mortality statistics, can benefit from a rapid assessment of the global loss of LE as well as HLE, relative to pre-pandemic values. These indicators can help to understand the impact of mortality on certain health conditions, or combinations of chronic diseases, enabling a precise allocation of resources and complex treatments from a population perspective, i.e. beyond the standard segmentations based on demographic characteristics or specific risk factors.

Throughout the study, we proposed a new approach to the concept of healthy life based on available data. The same perspective can be also applicable to other Health Expectancies based on surveys, for example disability-free Life (less subjective and more related to legal or country system characteristics), without chronic morbidity or active life health expectancies.

Concerning the external validation of the results, the same model and data processing shown in the Figs 2 and 3 can be transferred to different contexts of healthcare services provision or geographical areas. However, two aspects must be considered. First, there is no international consensus on patient classification systems from a global perspective. In this work we used the 3M™ Clinical Risk Groups (CRG) [15], but a well-known study of the Society of Actuaries compared 12 different diagnosis and/or pharmacy based models [18]. A second concern is related to the quality of the data. Regardless of the particular system used, the accuracy of the patient classification depends on the richness and intensity of ICD and ATC codes. A poor level of patient-episode data will certainly result in a biased patient classification. This is probably the most controversial point of the proposed methodology and contrasts with the broad consensus and comparability of the Sullivan method.

### Limitations

Our work is focused on the production of health indicators using data, and some particular issues related to mortality estimation leave room for improvement. This orientation can be considered as a limitation of the work. That is the case of the general age-specific mortality estimation, field in which research is in constant development [37], but in particular at the extremes of life [38–40].

Finally, as a proposal for further research, a refined version of the model could be obtained by improving mortality and LE forecasting [41]. A refined version of the model could improve the precision of the estimates of LE at advanced ages shown in the Table 2 (HLE-MMS_90_).

## Conclusions

According to the results, health indicators such as HLE can be efficiently obtained from multistate models based on mortality and morbidity information, without the use of health surveys. The major implication is that health indicators could be more easily obtained and extensively used for monitoring sub-national populations according to their health status, in the context of health planning. Compared with the standard Sullivan method, this new alternative gains applicability at the expense of reducing comparability. LE results were consistent with the standard government reports. Due to the different approximation to the concept of health (data-based versus self-perception), healthy life expectancies obtained from multistate micro simulation are consistently lower than those calculated with the standard Sullivan method.

## Supporting information

S1 Table Study population at December 31, 2016. (PDF)

S2 Table Group-centred transition matrices. (PDF)

S3 Table Age-specific transition matrices. (PDF)

## References

[1] Siegel JS. Concepts and Basic Measures of Mortality. The Demography and Epidemiology of Human Health and Aging. Dordrecht: Springer Netherlands; 2012. pp. 73–134. doi: 10.1007/978-94-007-1315-4_3

[2] Gispert R, Puig X, Puigdefàbregas A, Tresserras R, Busquets E. Esperanza de vida libre de incapacidad y esperanza de vida en buena salud en Cataluña 1994–2000. Med Clin. 2003;121(Supl 1): 128–132.

[3] Pol LG, Thomas RK. The Demography of Health and Healthcare. Dordrecht: Springer Netherlands; 2013. doi: 10.1007/978-90-481-8903-8

[4] European Commission E. Final report of the expert group on quality of life indicators. 2017 p. 119. doi: 10.2785/021270

[5] Ministerio de Sanidad Servicios Sociales e Igualdad. Esperanzas de vida en España, 2013. Madrid; 2015. Avaliable from: https://www.sanidad.gob.es/estadEstudios/estadisticas/inforRecopilaciones/EsperanzasDeVida_2013.pdf

[6] Kong F. Aging trenf of the world. In: Hoshi T, Kodama S, editors. The Structure of Healthy Life Determinants Lessons from the Japanese Aging Cohort Studies. Singapore: Springer Singapore; 2018. pp. 7–21 pp. doi: 10.1007/978-981-10-6629-0

[7] Departament de Salut Generalitat de Catalunya. Pla de salut de Catalunya 2021–2025. Departament de Salut Generalitat de Catalunya, editor. Barcelona: Generalitat de Catalunya. Departament de Salut; 2021. Available: https://scientiasalut.gencat.cat/handle/11351/7948

[8] Siegel JS. The Demography and Epidemiology of Human Health and Aging. 2nd Ed. Igarss 2014. Dordrecht: Springer Netherlands; 2012. doi: 10.1007/978-94-007-1315-4

[9] Sullivan DF. A Single Index of Mortality and Morbidity. HSMHA Health Rep. 1971;86: 347. doi: 10.2307/4594169 5554262 PMC1937122

[10] Encuesta Nacional de Salud de España 2017. Ministerio de Sanidad. [cited 10 Jan 2024]. Avaliable from: https://www.sanidad.gob.es/estadEstudios/estadisticas/encuestaNacional/encuesta2017.htm

[11] Jagger C, Hauet E, Brouard N. Health Expectancy Calculation by the Sullivan Method: A Practical Guide. 2001. Report No.: REVES Paper n°408. Available: https://reves.site.ined.fr/fichier/s_rubrique/20184/rp408.fr.pdf

[12] Robine J-M, Cambois E, Nusselder W, Jeune B, Van Oyen H, Jagger C. The joint action on healthy life years (JA: EHLEIS). Arch Public Heal. 2013;71: 2. doi: 10.1186/0778-7367-71-2 23379576 PMC3598905

[13] Departament de Salut, Generalitat de Catalunya. Enquesta de salut de Catalunya (ESCA). [cited 10 Jan 2024]. Avaliable from: https://salutweb.gencat.cat/ca/departament/estadistiques-sanitaries/enquestes/esca/index.html

[14] Eurostat. EU statistics on income and living conditions (EU-SILC) methodology—economic strain. 2015; 1–3. Avaliable from: http://ec.europa.eu/eurostat/statistics-explained/index.php/EU_statistics_on_income_and_living_conditions_(EU-SILC)_methodology

[15] Hughes JS, Averill RF, Eisenhandler J, Goldfield NI, Muldoon J, Neff JM, et al. Clinical Risk Groups (CRGs): a classification system for risk-adjusted capitation-based payment and health care management. Med Care. 2004;42: 81–90. doi: 10.1097/01.mlr.0000102367.93252.70 14713742

[16] Inoriza JM, Coderch J, Carreras M, Vall-llosera L, García-Goñi M, Lisbona JM, et al. La medida de la morbilidad atendida en una organización sanitaria integrada. Gac Sanit. 2009;23: 29–37. doi: 10.1016/j.gaceta.2008.02.003 19231720

[17] Starfield B, Weiner J, Mumford L, Steinwachs D. Ambulatory care groups: a categorization of diagnoses for research and management. Health Serv Res. 1991;26: 53–74. Avaliable from: https://www.ncbi.nlm.nih.gov/pmc/articles/PMC1069810/ 1901841 PMC1069810

[18] Winkelman R, Mehmud S, Wachenheim L. A Comparative Analysis of Claims-Based Tools for Health Risk Assessment. 2007. Avaliable from: https://www.soa.org/resources/research-reports/2007/hlth-risk-assement/

[19] Krijkamp EM, Alarid-Escudero F, Enns EA, Jalal HJ, Hunink MGM, Pechlivanoglou P. Microsimulation Modeling for Health Decision Sciences Using R: A Tutorial. Med Decis Mak. 2018;38: 400–422. doi: 10.1177/0272989X18754513 29587047 PMC6349385

[20] Kirsch F. Economic Evaluations of Multicomponent Disease Management Programs with Markov Models: A Systematic Review. Value Heal. 2016;19: 1039–1054. doi: 10.1016/j.jval.2016.07.004 27987631

[21] Gilks W, Richardson S, Spiegelhalter D. Introducing Markov Chain MonteCarlo. 1st ed. In: Gilks WR, Richardson S, David Spiegelhalter, editors. Markov Chain Monte Carlo in Practice. 1st ed. London: Chapman and Hall; 1996. pp. 1–16.

[22] Gardiner C. Stochastic Methods: A Handbook for the Natural and Social Sciences: 13. 4th ed. Heidelberg: Spinger-Verlag; 2009. Avaliable from: https://link.springer.com/book/9783540707127

[23] Santos JV, Martins FS, Pestana J, Souza J, Freitas A, Cylus J. Should we adjust health expenditure for age structure on health systems efficiency? A worldwide analysis. Health Econ Rev. 2023;13: 11. doi: 10.1186/s13561-023-00421-2 36781709 PMC9926817

[24] Freitas-Ramírez A, Puigdefàbregas-Serra A, Ribas G, Molina P. Anàlisi de la mortalitat a Catalunya, 2017: resum de resultats. Butll Epidemiol Catalunya. 2019;40: 205–217. Avaliable from: https://hdl.handle.net/11351/5651

[25] Classficació Internacional de Malalties 9a Revisió Modificacio Clínica (CIM-9-MC). 8a edició. Barcelona: Servei Catala de la Salut; 2011. Avaliable from: https://hdl.handle.net/11351/6894

[26] Anatomical Therapeutic Chemical (ATC) Classification. In: World Health Organization. 2011. [cited 10 Jan 2024]. Avaliable from: https://www.who.int/tools/atc-ddd-toolkit/atc-classification

[27] Microsoft Visual Studio Docs. Office Visual Basic for Applications (VBA) reference | Microsoft Docs. [cited 10 Jan 2024]. Avaliable from: https://learn.microsoft.com/en-us/office/vba/api/overview/

[28] INE. Esperanza de vida a diferentes edades. In: Esperanza de vida de un hombre en España. 2021 [cited 10 Jan 2024]. Avaliable from: https://www.ine.es/ss/Satellite?L=es_ES&c=INESeccion_C&cid=1259944484459&p=1254735110672&pagename=ProductosYServicios%2FPYSLayout&param1=PYSDetalleFichaIndicador&param3=1259947308577

[29] van Druten VP, Bartels EA, van de Mheen D, de Vries E, Kerckhoffs APM, Nahar-van Venrooij LMW. Concepts of health in different contexts: a scoping review. BMC Health Serv Res. 2022;22: 389. doi: 10.1186/s12913-022-07702-2 35331223 PMC8953139

[30] Jylhä M. What is self-rated health and why does it predict mortality? Towards a unified conceptual model. Soc Sci Med. 2009;69: 307–316. doi: 10.1016/j.socscimed.2009.05.013 19520474

[31] Mannion C, Hughes J, Moriarty F, Bennett K, Cahir C. Agreement between self-reported morbidity and pharmacy claims data for prescribed medications in an older community based population. BMC Geriatr. 2020;20: 283. doi: 10.1186/s12877-020-01684-8 32778067 PMC7419222

[32] Richardson K, Kenny RA, Peklar J, Bennett K. Agreement between patient interview data on prescription medication use and pharmacy records in those aged older than 50 years varied by therapeutic group and reporting of indicated health conditions. J Clin Epidemiol. 2013;66: 1308–1316. doi: 10.1016/j.jclinepi.2013.02.016 23968693

[33] Carreras M, Puig G, Sánchez-Pérez I, Inoriza JM, Coderch J, Gispert R. Morbilidad y estado de salud autopercibido, dos aproximaciones diferentes al estado de salud. Gac Sanit. 2020;34: 601–607. doi: 10.1016/j.gaceta.2019.04.005 31255397

[34] Sarah B., Laditka, James N. Laditka CJ. Microsimulation of Health Expectancies, Life Course Health, and Health Policy Outcomes. International Handbook of Health Expectancies, 2020, Volume 9. 2020. pp. 129–139. Avaliable from: https://link.springer.com/chapter/10.1007/978-3-030-37668-0_9

[35] Laditka SB, Hayward MD. The Evolution of Demographic Methods to Calculate Health Expectancies. Determining Health Expectancies. Chichester, UK: John Wiley & Sons, Ltd; 2003. pp. 221–234. doi: 10.1002/0470858885.ch11

[36] Wang H, Paulson KR, Pease SA, Watson S, Comfort H, Zheng P, et al. Estimating excess mortality due to the COVID-19 pandemic: a systematic analysis of COVID-19-related mortality, 2020–21. Lancet. 2022;6736: 1–24. doi: 10.1016/s0140-6736(21)02796-3 35279232 PMC8912932

[37] Leontyeva Y, Bower H, Gauffin O, Lambert PC, Andersson TML. Assessing the impact of including variation in general population mortality on standard errors of relative survival and loss in life expectancy. BMC Med Res Methodol. 2022;22: 1–15. doi: 10.1186/s12874-022-01597-7 35501701 PMC9059421

[38] Perin J, Chu Y, Villavicencio F, Schumacher A, McCormick T, Guillot M, et al. Adapting and validating the log quadratic model to derive under-five age- and cause-specific mortality (U5ACSM): a preliminary analysis. Popul Health Metr. 2022;20: 1–12. doi: 10.1186/s12963-021-00277-w 35012587 PMC8744238

[39] Gavrilova NS, Gavrilov LA, Krut’ko VN. Mortality Trajectories at Exceptionally High Ages: A Study of Supercentenarians. Living to 100 Monogr. 2017;2017: 139–148. Avaliable from: https://pubmed.ncbi.nlm.nih.gov/29170764/PMC5696798 29170764

[40] Alvarez J-A, Villavicencio F, Strozza C, Camarda CG. Regularities in human mortality after age 105. Lanza Queiroz B, editor. PLoS One. 2021;16: e0253940. doi: 10.1371/journal.pone.0253940 34260647 PMC8279393

[41] Shang HL. Statistically tested comparisons of the accuracy of forecasting methods for age-specific and sex-specific mortality and life expectancy. Popul Stud (NY). 2015;69: 317–335. doi: 10.1080/00324728.2015.1074268 26452306

