# Supplementary material for: Measuring population health using health expectancy estimates from morbidity and mortality databases
Source: PLoS One. 2024 May 21;19(5):e0302174. doi: 10.1371/journal.pone.0302174 (PMC11108136; doi:10.1371/journal.pone.0302174)
Supplement: S1 Table — (PDF) [file pone.0302174.s001.pdf]

S1 Table. Study population at December 31, 2016

	Healthy		Acute		Minor chronic		Major chronic		Major chronic >= 3, catastrophic		Metastatic malignacies	
	Male	Female	Male	Female	Male	Female	Male	Female	Male	Female	Male	Female
0-1	348	342	64	38	11	8	15	13	0	0	0	0
1-14	5,217	4,952	762	650	434	378	648	536	4	7	2	3
15-24	3,445	2,832	371	459	254	396	318	346	3	3	2	2
25-34	3,703	2,942	419	845	364	783	408	425	7	5	4	2
35-44	4,933	3,689	654	867	815	1,396	1,054	1,003	23	17	12	8
45-54	3,431	2,612	470	464	827	1,570	2,109	1,862	72	46	36	23
55-64	1,581	1,420	214	235	599	1,140	2,809	2,495	104	73	52	37
65-74	505	544	62	74	265	655	2,864	2,821	177	113	89	57
75-84	141	164	9	30	80	201	1,755	2,366	242	186	121	93
>=85	38	87	6	10	24	70	683	1,388	105	167	53	83
