# Supplementary material for: Measuring population health using health expectancy estimates from morbidity and mortality databases
Source: PLoS One. 2024 May 21;19(5):e0302174. doi: 10.1371/journal.pone.0302174 (PMC11108136; doi:10.1371/journal.pone.0302174)
Supplement: S2 Table — (PDF) [file pone.0302174.s002.pdf]

S2 Table. Transition probabilities by age group, from health status i at age t-1 to health status j at age t

Age' t-1' and status 'i' in rows, age 't' and status 'j' in columns.
1: Healthy, 2: Significant acute disease, 3: Minor chronic disease, 4: Significant chronic disease in one or two organ systems, 5: Significant chronic disease in three or more organ systems - Catastrophic conditions, 6: Dominant and metastatic malignancies, 7: Death.

Male

Age <=1	1	2	3	4	5	6	7
1	0.6896	0.1983	0.0517	0.0575	0.0029	0.0000	0.0000
2	0.5468	0.2969	0.0625	0.0938	0.0000	0.0000	0.0000
3	0.2727	0.0909	0.4545	0.1818	0.0000	0.0000	0.0000
4	0.1333	0.2667	0.0667	0.5333	0.0000	0.0000	0.0000
5	0.0000	0.0000	0.0000	0.0000	0.0000	0.0000	0.0000
6	0.0000	0.0000	0.0000	0.0000	0.0000	0.0000	0.0000
7	0.0000	0.0000	0.0000	0.0000	0.0000	0.0000	1.0000

Age 1-14	1	2	3	4	5	6	7
1	0.8324	0.0861	0.0362	0.0447	0.0006	0.0000	0.0000
2	0.6391	0.1929	0.0840	0.0840	0.0000	0.0000	0.0000
3	0.5368	0.1014	0.2581	0.1014	0.0023	0.0000	0.0000
4	0.3118	0.0802	0.0571	0.5479	0.0015	0.0015	0.0000
5	0.0000	0.0000	0.0000	0.0000	1.0000	0.0000	0.0000
6	0.0000	0.0000	0.0000	0.5000	0.0000	0.5000	0.0000
7	0.0000	0.0000	0.0000	0.0000	0.0000	0.0000	1.0000

Age 15-24	1	2	3	4	5	6	7
1	0.8740	0.0615	0.0372	0.0270	0.0003	0.0000	0.0000
2	0.7251	0.1563	0.0701	0.0485	0.0000	0.0000	0.0000
3	0.5355	0.1142	0.2913	0.0551	0.0000	0.0039	0.0000
4	0.3648	0.0503	0.0346	0.5472	0.0000	0.0000	0.0031
5	0.0000	0.0000	0.0000	0.0000	1.0000	0.0000	0.0000
6	0.0000	0.0000	0.0000	0.5000	0.0000	0.5000	0.0000
7	0.0000	0.0000	0.0000	0.0000	0.0000	0.0000	1.0000

Age 25-34	1	2	3	4	5	6	7
1	0.8373	0.0778	0.0516	0.0327	0.0000	0.0003	0.0003
2	0.6540	0.1766	0.1026	0.0668	0.0000	0.0000	0.0000
3	0.4506	0.1236	0.3517	0.0714	0.0027	0.0000	0.0000
4	0.2083	0.0490	0.0686	0.6716	0.0000	0.0025	0.0000
5	0.0000	0.0000	0.0000	0.1111	0.7778	0.0000	0.1111
6	0.0000	0.0000	0.0000	0.0000	0.0000	1.0000	0.0000
7	0.0000	0.0000	0.0000	0.0000	0.0000	0.0000	1.0000

Age 35-44	1	2	3	4	5	6	7
1	0.8189	0.0801	0.0574	0.0432	0.0000	0.0002	0.0002
2	0.6009	0.1957	0.1239	0.0795	0.0000	0.0000	0.0000
3	0.3988	0.0736	0.4184	0.1092	0.0000	0.0000	0.0000
4	0.1841	0.0465	0.0825	0.6774	0.0076	0.0000	0.0019
5	0.1000	0.0000	0.0000	0.1000	0.8000	0.0000	0.0000
6	0.2000	0.0000	0.0000	0.0000	0.0000	0.6000	0.2000
7	0.0000	0.0000	0.0000	0.0000	0.0000	0.0000	1.0000

Age 45-54	1	2	3	4	5	6	7
1	0.7854	0.0694	0.0679	0.0749	0.0009	0.0003	0.0012
2	0.5383	0.1830	0.1106	0.1638	0.0000	0.0043	0.0000
3	0.2757	0.0810	0.4655	0.1753	0.0000	0.0000	0.0024
4	0.1024	0.0237	0.0597	0.8005	0.0066	0.0038	0.0033
5	0.0111	0.0111	0.0000	0.0333	0.9223	0.0000	0.0222
6	0.0000	0.0000	0.0556	0.1111	0.0000	0.6111	0.2222
7	0.0000	0.0000	0.0000	0.0000	0.0000	0.0000	1.0000

Age 55-64	1	2	3	4	5	6	7
1	0.7368	0.0633	0.0772	0.1139	0.0032	0.0025	0.0032
2	0.4579	0.1729	0.1495	0.2103	0.0000	0.0047	0.0047
3	0.2087	0.0601	0.5074	0.2204	0.0000	0.0017	0.0017
4	0.0488	0.0100	0.0381	0.8782	0.0128	0.0046	0.0075
5	0.0000	0.0000	0.0000	0.2793	0.6667	0.0180	0.0360
6	0.0222	0.0000	0.0444	0.1778	0.0444	0.5779	0.1333
7	0.0000	0.0000	0.0000	0.0000	0.0000	0.0000	1.0000

Age 65-74	1	2	3	4	5	6	7
1	0.6832	0.0515	0.0851	0.1683	0.0040	0.0079	0.0000
2	0.3871	0.2419	0.1774	0.1774	0.0000	0.0000	0.0161
3	0.1208	0.0566	0.5094	0.3094	0.0000	0.0000	0.0038
4	0.0203	0.0045	0.0258	0.8984	0.0272	0.0133	0.0105
5	0.0000	0.0000	0.0058	0.2500	0.6686	0.0000	0.0756
6	0.0213	0.0106	0.0213	0.1383	0.0106	0.5639	0.2340
7	0.0000	0.0000	0.0000	0.0000	0.0000	0.0000	1.0000

Age 75-84	1	2	3	4	5	6	7
1	0.6737	0.0284	0.0638	0.2199	0.0000	0.0000	0.0142
2	0.2222	0.2222	0.2222	0.3333	0.0000	0.0000	0.0000
3	0.1500	0.0000	0.5125	0.3375	0.0000	0.0000	0.0000
4	0.0137	0.0023	0.0120	0.8666	0.0479	0.0205	0.0370
5	0.0000	0.0000	0.0000	0.2263	0.6461	0.0206	0.1070
6	0.0083	0.0000	0.0000	0.1750	0.0500	0.5417	0.2250
7	0.0000	0.0000	0.0000	0.0000	0.0000	0.0000	1.0000

Age >= 85	1	2	3	4	5	6	7
1	0.6316	0.0000	0.0263	0.2632	0.0263	0.0000	0.0526
2	0.1667	0.1667	0.3333	0.3333	0.0000	0.0000	0.0000
3	0.1250	0.0000	0.3333	0.5000	0.0000	0.0000	0.0417
4	0.0132	0.0029	0.0088	0.7671	0.0630	0.0205	0.1245
5	0.0000	0.0000	0.0000	0.2328	0.5258	0.0086	0.2328
6	0.0238	0.0000	0.0000	0.1667	0.0238	0.4048	0.3810
7	0.0000	0.0000	0.0000	0.0000	0.0000	0.0000	1.0000

Female

Age <=1	1	2	3	4	5	6	7
1	0.7485	0.1550	0.0468	0.0468	0.0000	0.0000	0.0029
2	0.5263	0.2895	0.0526	0.1316	0.0000	0.0000	0.0000
3	0.3750	0.1250	0.3750	0.1250	0.0000	0.0000	0.0000
4	0.4167	0.0833	0.1667	0.3333	0.0000	0.0000	0.0000
5	0.0000	0.0000	0.0000	1.0000	0.0000	0.0000	0.0000
6	0.0000	0.0000	0.0000	0.0000	0.0000	0.0000	0.0000
7	0.0000	0.0000	0.0000	0.0000	0.0000	0.0000	1.0000

Age 1-14	1	2	3	4	5	6	7
1	0.8394	0.0824	0.0400	0.0378	0.0002	0.0002	0.0000
2	0.6185	0.1985	0.0877	0.0938	0.0015	0.0000	0.0000
3	0.5634	0.0979	0.2302	0.1085	0.0000	0.0000	0.0000
4	0.3619	0.0653	0.0522	0.5206	0.0000	0.0000	0.0000
5	0.1250	0.0000	0.0000	0.1250	0.7500	0.0000	0.0000
6	0.0000	0.0000	0.0000	0.5000	0.0000	0.5000	0.0000
7	0.0000	0.0000	0.0000	0.0000	0.0000	0.0000	1.0000

Age 15-24	1	2	3	4	5	6	7
1	0.8276	0.0925	0.0537	0.0258	0.0004	0.0000	0.0000
2	0.6143	0.2092	0.1133	0.0610	0.0000	0.0000	0.0022
3	0.4596	0.1465	0.3485	0.0455	0.0000	0.0000	0.0000
4	0.3179	0.0896	0.1069	0.4855	0.0000	0.0000	0.0000
5	0.0000	0.0000	0.0000	0.2000	0.8000	0.0000	0.0000
6	0.0000	0.0000	0.0000	0.0000	0.0000	0.0000	0.0000
7	0.0000	0.0000	0.0000	0.0000	0.0000	0.0000	1.0000

Age 25-34	1	2	3	4	5	6	7
1	0.7482	0.1312	0.0877	0.0326	0.0000	0.0000	0.0003
2	0.5409	0.2426	0.1396	0.0757	0.0000	0.0012	0.0000
3	0.2950	0.1801	0.4572	0.0677	0.0000	0.0000	0.0000
4	0.2235	0.1247	0.1482	0.5012	0.0024	0.0000	0.0000
5	0.0000	0.0000	0.0000	0.2000	0.8000	0.0000	0.0000
6	0.5000	0.0000	0.0000	0.0000	0.0000	0.5000	0.0000
7	0.0000	0.0000	0.0000	0.0000	0.0000	0.0000	1.0000

Age 35-44	1	2	3	4	5	6	7
1	0.7563	0.1044	0.0992	0.0390	0.0000	0.0008	0.0003
2	0.5202	0.2099	0.1822	0.0877	0.0000	0.0000	0.0000
3	0.2629	0.1067	0.5380	0.0917	0.0007	0.0000	0.0000
4	0.1476	0.0469	0.1505	0.6490	0.0020	0.0020	0.0020
5	0.0000	0.0000	0.0000	0.1250	0.8750	0.0000	0.0000
6	0.2222	0.1111	0.0000	0.2222	0.0000	0.4444	0.0000
7	0.0000	0.0000	0.0000	0.0000	0.0000	0.0000	1.0000

Age 45-54	1	2	3	4	5	6	7
1	0.7614	0.0800	0.0992	0.0586	0.0004	0.0004	0.0000
2	0.4138	0.2026	0.2349	0.1487	0.0000	0.0000	0.0000
3	0.1847	0.0643	0.6001	0.1484	0.0006	0.0013	0.0006
4	0.0806	0.0306	0.1117	0.7680	0.0043	0.0016	0.0032
5	0.0000	0.0000	0.0238	0.1429	0.8333	0.0000	0.0000
6	0.0741	0.0370	0.0370	0.1481	0.0000	0.6668	0.0370
7	0.0000	0.0000	0.0000	0.0000	0.0000	0.0000	1.0000

Age 55-64	1	2	3	4	5	6	7
1	0.7323	0.0613	0.0944	0.1099	0.0000	0.0007	0.0014
2	0.4000	0.2000	0.2255	0.1745	0.0000	0.0000	0.0000
3	0.1246	0.0447	0.6299	0.1982	0.0000	0.0026	0.0000
4	0.0449	0.0112	0.0946	0.8337	0.0080	0.0056	0.0020
5	0.0339	0.0000	0.0169	0.3051	0.6102	0.0000	0.0339
6	0.0784	0.0000	0.0980	0.2941	0.0000	0.4510	0.0784
7	0.0000	0.0000	0.0000	0.0000	0.0000	0.0000	1.0000

Age 65-74	1	2	3	4	5	6	7
1	0.7225	0.0496	0.1029	0.1176	0.0000	0.0037	0.0037
2	0.3649	0.1486	0.2838	0.2027	0.0000	0.0000	0.0000
3	0.0687	0.0290	0.6366	0.2595	0.0000	0.0031	0.0031
4	0.0181	0.0050	0.0645	0.8911	0.0131	0.0043	0.0039
5	0.0096	0.0000	0.0000	0.2115	0.6828	0.0096	0.0865
6	0.0000	0.0000	0.0303	0.1970	0.0000	0.6515	0.1212
7	0.0000	0.0000	0.0000	0.0000	0.0000	0.0000	1.0000

Age 75-84	1	2	3	4	5	6	7
1	0.6951	0.0549	0.0610	0.1463	0.0061	0.0000	0.0366
2	0.3000	0.1000	0.1000	0.4333	0.0000	0.0000	0.0667
3	0.0547	0.0100	0.6069	0.3035	0.0050	0.0000	0.0199
4	0.0156	0.0021	0.0270	0.8940	0.0338	0.0076	0.0199
5	0.0089	0.0000	0.0000	0.2321	0.6697	0.0000	0.0893
6	0.0000	0.0000	0.0182	0.2000	0.0000	0.6000	0.1818
7	0.0000	0.0000	0.0000	0.0000	0.0000	0.0000	1.0000

Age >= 85	1	2	3	4	5	6	7
1	0.6436	0.0345	0.0345	0.2069	0.0000	0.0000	0.0805
2	0.1000	0.0000	0.1000	0.6000	0.0000	0.0000	0.2000
3	0.0286	0.0000	0.6143	0.2857	0.0143	0.0000	0.0571
4	0.0173	0.0007	0.0137	0.7904	0.0612	0.0014	0.1153
5	0.0087	0.0000	0.0043	0.1905	0.5757	0.0000	0.2208
6	0.0526	0.0000	0.0000	0.1053	0.0000	0.6316	0.2105
7	0.0000	0.0000	0.0000	0.0000	0.0000	0.0000	1.0000
