# Supplementary material for: Measuring population health using health expectancy estimates from morbidity and mortality databases
Source: PLoS One. 2024 May 21;19(5):e0302174. doi: 10.1371/journal.pone.0302174 (PMC11108136; doi:10.1371/journal.pone.0302174)
Supplement: S3 Table — (PDF) [file pone.0302174.s003.pdf]

S3 Table. Yearly transition probabilities, from health status i at age t-1 to health status j at age t
(Cubic Spline interpolation)

Age' t-1' and status 'i' in rows, age 't' and status 'j' in columns.
1: Healthy, 2: Significant acute disease, 3: Minor chronic disease, 4: Significant chronic disease in one or two organ systems, 5: Significant chronic disease in three or more organ systems - Catastrophic conditions, 6: Dominant and metastatic malignancies, 7: Death.

Male

Age 0	1	2	3	4	5	6	7
1	0.6896	0.1983	0.0517	0.0575	0.0029	0.0000	0.0000
2	0.5468	0.2969	0.0625	0.0938	0.0000	0.0000	0.0000
3	0.2727	0.0909	0.4545	0.1818	0.0000	0.0000	0.0000
4	0.1333	0.2667	0.0667	0.5333	0.0000	0.0000	0.0000
5	0.0000	0.0000	0.0000	0.0000	0.0000	0.0000	0.0000
6	0.0000	0.0000	0.0000	0.0000	0.0000	0.0000	0.0000
7	0.0000	0.0000	0.0000	0.0000	0.0000	0.0000	1.0000

Age 1	1	2	3	4	5	6	7
1	0.8027	0.1028	0.0381	0.0555	0.0007	0.0001	0.0001
2	0.5795	0.2164	0.0964	0.1077	0.0000	0.0000	0.0000
3	0.5220	0.0985	0.2461	0.1291	0.0043	0.0000	0.0000
4	0.2620	0.0784	0.0632	0.5912	0.0020	0.0032	0.0000
5	0.0000	0.0000	0.0005	0.0078	0.9911	0.0000	0.0006
6	0.0000	0.0000	0.0000	0.0000	0.0000	0.0000	0.0000
7	0.0000	0.0000	0.0000	0.0000	0.0000	0.0000	1.0000

Age 2	1	2	3	4	5	6	7
1	0.8070	0.1004	0.0380	0.0539	0.0007	0.0000	0.0000
2	0.5881	0.2129	0.0947	0.1043	0.0000	0.0000	0.0000
3	0.5236	0.0992	0.2483	0.1249	0.0040	0.0000	0.0000
4	0.2683	0.0780	0.0629	0.5860	0.0019	0.0029	0.0000
5	0.0000	0.0000	0.0005	0.0078	0.9911	0.0000	0.0006
6	0.0000	0.0000	0.0000	0.0000	0.0000	0.0000	0.0000
7	0.0000	0.0000	0.0000	0.0000	0.0000	0.0000	1.0000

Age 3	1	2	3	4	5	6	7
1	0.8117	0.0977	0.0377	0.0522	0.0007	0.0000	0.0000
2	0.5974	0.2092	0.0929	0.1005	0.0000	0.0000	0.0000
3	0.5257	0.0997	0.2504	0.1205	0.0037	0.0000	0.0000
4	0.2758	0.0776	0.0626	0.5795	0.0018	0.0027	0.0000
5	0.0000	0.0000	0.0004	0.0078	0.9913	0.0000	0.0005
6	0.0000	0.0000	0.0000	0.0000	0.0000	0.0000	0.0000
7	0.0000	0.0000	0.0000	0.0000	0.0000	0.0000	1.0000

Age 4	1	2	3	4	5	6	7
1	0.8166	0.0950	0.0374	0.0504	0.0006	0.0000	0.0000
2	0.6073	0.2053	0.0908	0.0966	0.0000	0.0000	0.0000
3	0.5281	0.1002	0.2524	0.1159	0.0034	0.0000	0.0000
4	0.2841	0.0772	0.0622	0.5723	0.0018	0.0024	0.0000
5	0.0000	0.0000	0.0003	0.0078	0.9915	0.0000	0.0004
6	0.0000	0.0000	0.0000	0.0000	0.0000	0.0000	0.0000
7	0.0000	0.0000	0.0000	0.0000	0.0000	0.0000	1.0000

Age 5	1	2	3	4	5	6	7
1	0.8218	0.0921	0.0370	0.0485	0.0006	0.0000	0.0000
2	0.6176	0.2013	0.0886	0.0925	0.0000	0.0000	0.0000
3	0.5307	0.1007	0.2544	0.1112	0.0030	0.0000	0.0000
4	0.2931	0.0767	0.0619	0.5645	0.0017	0.0021	0.0000
5	0.0000	0.0000	0.0002	0.0077	0.9918	0.0000	0.0003
6	0.0000	0.0000	0.0000	0.0000	0.0000	0.0000	0.0000
7	0.0000	0.0000	0.0000	0.0000	0.0000	0.0000	1.0000

Age 6	1	2	3	4	5	6	7
1	0.8271	0.0891	0.0366	0.0466	0.0006	0.0000	0.0000
2	0.6283	0.1971	0.0863	0.0883	0.0000	0.0000	0.0000
3	0.5338	0.1010	0.2562	0.1063	0.0027	0.0000	0.0000
4	0.3004	0.0831	0.0604	0.5527	0.0016	0.0018	0.0000
5	0.0000	0.0000	0.0002	0.0077	0.9919	0.0000	0.0002
6	0.0000	0.0000	0.0000	0.0000	0.0000	0.0000	0.0000
7	0.0000	0.0000	0.0000	0.0000	0.0000	0.0000	1.0000

Age 7	1	2	3	4	5	6	7
1	0.8324	0.0861	0.0362	0.0447	0.0006	0.0000	0.0000
2	0.6391	0.1929	0.0840	0.0840	0.0000	0.0000	0.0000
3	0.5368	0.1014	0.2581	0.1014	0.0023	0.0000	0.0000
4	0.3118	0.0802	0.0571	0.5479	0.0015	0.0015	0.0000
5	0.0000	0.0000	0.0001	0.0026	0.9972	0.0000	0.0001
6	0.0000	0.0000	0.0000	0.0000	0.0000	0.0000	0.0000
7	0.0000	0.0000	0.0000	0.0000	0.0000	0.0000	1.0000

Age 8	1	2	3	4	5	6	7
1	0.8379	0.0830	0.0358	0.0427	0.0006	0.0000	0.0000
2	0.6499	0.1887	0.0817	0.0797	0.0000	0.0000	0.0000
3	0.5397	0.1017	0.2598	0.0964	0.0019	0.0005	0.0000
4	0.3229	0.0773	0.0538	0.5427	0.0015	0.0013	0.0005

Female

Age 0	1	2	3	4	5	6	7
1	0.7485	0.1550	0.0468	0.0468	0.0000	0.0000	0.0029
2	0.5263	0.2895	0.0526	0.1316	0.0000	0.0000	0.0000
3	0.3750	0.1250	0.3750	0.1250	0.0000	0.0000	0.0000
4	0.4167	0.0833	0.1667	0.3333	0.0000	0.0000	0.0000
5	0.0000	0.0000	0.0000	1.0000	0.0000	0.0000	0.0000
6	0.0000	0.0000	0.0000	0.0000	0.0000	0.0000	0.0000
7	0.0000	0.0000	0.0000	0.0000	0.0000	0.0000	1.0000

Age 1	1	2	3	4	5	6	7
1	0.8312	0.0849	0.0378	0.0456	0.0001	0.0003	0.0001
2	0.6062	0.1998	0.0762	0.1151	0.0024	0.0003	0.0000
3	0.5939	0.0798	0.1786	0.1477	0.0000	0.0000	0.0000
4	0.3733	0.0620	0.0279	0.5363	0.0005	0.0000	0.0000
5	0.1962	0.0000	0.0003	0.0859	0.7176	0.0000	0.0000
6	0.0593	0.0000	0.0001	0.4384	0.0000	0.5020	0.0002
7	0.0000	0.0000	0.0000	0.0000	0.0000	0.0000	1.0000

Age 2	1	2	3	4	5	6	7
1	0.8318	0.0850	0.0384	0.0444	0.0001	0.0003	0.0000
2	0.6073	0.2000	0.0781	0.1120	0.0023	0.0003	0.0000
3	0.5875	0.0832	0.1874	0.1419	0.0000	0.0000	0.0000
4	0.3708	0.0631	0.0320	0.5337	0.0004	0.0000	0.0000
5	0.1853	0.0000	0.0003	0.0921	0.7223	0.0000	0.0000
6	0.0569	0.0000	0.0001	0.4409	0.0000	0.5019	0.0002
7	0.0000	0.0000	0.0000	0.0000	0.0000	0.0000	1.0000

Age 3	1	2	3	4	5	6	7
1	0.8328	0.0848	0.0389	0.0432	0.0001	0.0002	0.0000
2	0.6090	0.2000	0.0801	0.1086	0.0021	0.0002	0.0000
3	0.5819	0.0863	0.1961	0.1357	0.0000	0.0000	0.0000
4	0.3686	0.0639	0.0361	0.5310	0.0004	0.0000	0.0000
5	0.1738	0.0000	0.0002	0.0985	0.7275	0.0000	0.0000
6	0.0521	0.0000	0.0001	0.4459	0.0000	0.5017	0.0002
7	0.0000	0.0000	0.0000	0.0000	0.0000	0.0000	1.0000

Age 4	1	2	3	4	5	6	7
1	0.8341	0.0844	0.0393	0.0419	0.0001	0.0002	0.0000
2	0.6109	0.1998	0.0820	0.1051	0.0020	0.0002	0.0000
3	0.5769	0.0893	0.2047	0.1291	0.0000	0.0000	0.0000
4	0.3667	0.0644	0.0402	0.5284	0.0003	0.0000	0.0000
5	0.1620	0.0000	0.0002	0.1050	0.7328	0.0000	0.0000
6	0.0445	0.0000	0.0001	0.4538	0.0000	0.5014	0.0002
7	0.0000	0.0000	0.0000	0.0000	0.0000	0.0000	1.0000

Age 5	1	2	3	4	5	6	7
1	0.8358	0.0838	0.0396	0.0405	0.0001	0.0002	0.0000
2	0.6134	0.1994	0.0839	0.1014	0.0018	0.0001	0.0000
3	0.5722	0.0922	0.2132	0.1224	0.0000	0.0000	0.0000
4	0.3650	0.0648	0.0442	0.5258	0.0002	0.0000	0.0000
5	0.1498	0.0000	0.0001	0.1116	0.7385	0.0000	0.0000
6	0.0338	0.0000	0.0001	0.4649	0.0000	0.5011	0.0001
7	0.0000	0.0000	0.0000	0.0000	0.0000	0.0000	1.0000

Age 6	1	2	3	4	5	6	7
1	0.8375	0.0831	0.0398	0.0392	0.0002	0.0002	0.0000
2	0.6157	0.1990	0.0858	0.0977	0.0017	0.0001	0.0000
3	0.5677	0.0951	0.2217	0.1155	0.0000	0.0000	0.0000
4	0.3634	0.0651	0.0482	0.5232	0.0001	0.0000	0.0000
5	0.1375	0.0000	0.0001	0.1183	0.7441	0.0000	0.0000
6	0.0193	0.0000	0.0000	0.4800	0.0000	0.5006	0.0001
7	0.0000	0.0000	0.0000	0.0000	0.0000	0.0000	1.0000

Age 7	1	2	3	4	5	6	7
1	0.8394	0.0824	0.0400	0.0378	0.0002	0.0002	0.0000
2	0.6185	0.1985	0.0877	0.0938	0.0015	0.0000	0.0000
3	0.5634	0.0979	0.2302	0.1085	0.0000	0.0000	0.0000
4	0.3619	0.0653	0.0522	0.5206	0.0000	0.0000	0.0000
5	0.1250	0.0000	0.0000	0.1250	0.7500	0.0000	0.0000
6	0.0000	0.0000	0.0000	0.5000	0.0000	0.5000	0.0000
7	0.0000	0.0000	0.0000	0.0000	0.0000	0.0000	1.0000

Age 8	1	2	3	4	5	6	7
1	0.8413	0.0817	0.0402	0.0364	0.0002	0.0002	0.0000
2	0.6208	0.1979	0.0896	0.0900	0.0014	0.0000	0.0003
3	0.5592	0.1007	0.2386	0.1015	0.0000	0.0000	0.0000
4	0.3604	0.0655	0.0562	0.5179	0.0000	0.0000	0.0000

5	0.0001	0.0000	0.0000	0.0000	0.9999	0.0000	0.0000
6	0.0000	0.0000	0.0000	0.0000	0.0000	0.0000	0.0000
7	0.0000	0.0000	0.0000	0.0000	0.0000	0.0000	1.0000

Age 9	1	2	3	4	5	6	7
1	0.8432	0.0801	0.0354	0.0408	0.0005	0.0000	0.0000
2	0.6605	0.1846	0.0794	0.0755	0.0000	0.0000	0.0000
3	0.5423	0.1020	0.2615	0.0915	0.0016	0.0011	0.0000
4	0.3339	0.0744	0.0505	0.5379	0.0014	0.0010	0.0009
5	0.0003	0.0000	0.0000	0.0000	0.9997	0.0000	0.0000
6	0.0000	0.0000	0.0000	0.0000	0.0000	0.0000	0.0000
7	0.0000	0.0000	0.0000	0.0000	0.0000	0.0000	1.0000

Age 10	1	2	3	4	5	6	7
1	0.8483	0.0772	0.0350	0.0390	0.0005	0.0000	0.0000
2	0.6709	0.1805	0.0772	0.0714	0.0000	0.0000	0.0000
3	0.5448	0.1024	0.2633	0.0867	0.0012	0.0016	0.0000
4	0.3443	0.0716	0.0473	0.5334	0.0014	0.0007	0.0013
5	0.0005	0.0000	0.0000	0.0000	0.9995	0.0000	0.0000
6	0.0000	0.0000	0.0000	0.0000	0.0000	0.0000	0.0000
7	0.0000	0.0000	0.0000	0.0000	0.0000	0.0000	1.0000

Age 11	1	2	3	4	5	6	7
1	0.8532	0.0744	0.0347	0.0372	0.0005	0.0000	0.0000
2	0.6809	0.1766	0.0751	0.0674	0.0000	0.0000	0.0000
3	0.5470	0.1028	0.2652	0.0820	0.0009	0.0021	0.0000
4	0.3540	0.0688	0.0443	0.5294	0.0013	0.0005	0.0017
5	0.0007	0.0000	0.0000	0.0000	0.9993	0.0000	0.0000
6	0.0000	0.0000	0.0000	0.0000	0.0000	0.0000	0.0000
7	0.0000	0.0000	0.0000	0.0000	0.0000	0.0000	1.0000

Age 12	1	2	3	4	5	6	7
1	0.8578	0.0718	0.0345	0.0354	0.0005	0.0000	0.0000
2	0.6902	0.1729	0.0732	0.0637	0.0000	0.0000	0.0000
3	0.5488	0.1033	0.2672	0.0775	0.0006	0.0026	0.0000
4	0.3626	0.0661	0.0415	0.5263	0.0012	0.0002	0.0021
5	0.0008	0.0000	0.0000	0.0000	0.9992	0.0000	0.0000
6	0.0000	0.0000	0.0000	0.0000	0.0000	0.0000	0.0000
7	0.0000	0.0000	0.0000	0.0000	0.0000	0.0000	1.0000

Age 13	1	2	3	4	5	6	7
1	0.8620	0.0694	0.0343	0.0338	0.0005	0.0000	0.0000
2	0.6986	0.1695	0.0716	0.0603	0.0000	0.0000	0.0000
3	0.5501	0.1040	0.2693	0.0733	0.0003	0.0030	0.0000
4	0.3700	0.0635	0.0391	0.5238	0.0011	0.0000	0.0025
5	0.0009	0.0000	0.0000	0.0000	0.9991	0.0000	0.0000
6	0.0000	0.0000	0.0000	0.0000	0.0000	0.0000	0.0000
7	0.0000	0.0000	0.0000	0.0000	0.0000	0.0000	1.0000

Age 14	1	2	3	4	5	6	7
1	0.8658	0.0672	0.0343	0.0323	0.0004	0.0000	0.0000
2	0.7064	0.1663	0.0702	0.0571	0.0000	0.0000	0.0000
3	0.5507	0.1048	0.2717	0.0694	0.0001	0.0033	0.0000
4	0.3758	0.0610	0.0369	0.5225	0.0010	0.0000	0.0028
5	0.0010	0.0000	0.0000	0.0000	0.9990	0.0000	0.0000
6	0.0000	0.0000	0.0000	0.0000	0.0000	0.0000	0.0000
7	0.0000	0.0000	0.0000	0.0000	0.0000	0.0000	1.0000

Age 15	1	2	3	4	5	6	7
1	0.8690	0.0653	0.0344	0.0309	0.0004	0.0000	0.0000
2	0.7130	0.1635	0.0691	0.0544	0.0000	0.0000	0.0000
3	0.5506	0.1058	0.2742	0.0658	0.0000	0.0036	0.0000
4	0.3799	0.0587	0.0352	0.5223	0.0009	0.0000	0.0030
5	0.0010	0.0000	0.0000	0.0000	0.9990	0.0000	0.0000
6	0.0000	0.0000	0.0000	0.0000	0.0000	0.0000	0.0000
7	0.0000	0.0000	0.0000	0.0000	0.0000	0.0000	1.0000

Age 16	1	2	3	4	5	6	7
1	0.8715	0.0637	0.0346	0.0298	0.0004	0.0000	0.0000
2	0.7184	0.1611	0.0684	0.0521	0.0000	0.0000	0.0000
3	0.5494	0.1070	0.2770	0.0627	0.0000	0.0039	0.0000
4	0.3819	0.0566	0.0339	0.5237	0.0007	0.0000	0.0032
5	0.0009	0.0000	0.0000	0.0000	0.9991	0.0000	0.0000
6	0.0000	0.0000	0.0000	0.0000	0.0000	0.0000	0.0000
7	0.0000	0.0000	0.0000	0.0000	0.0000	0.0000	1.0000

Age 17	1	2	3	4	5	6	7
1	0.8735	0.0625	0.0349	0.0287	0.0004	0.0000	0.0000
2	0.7225	0.1591	0.0681	0.0503	0.0000	0.0000	0.0000
3	0.5475	0.1084	0.2801	0.0600	0.0000	0.0040	0.0000
4	0.3816	0.0547	0.0332	0.5266	0.0006	0.0000	0.0033
5	0.0008	0.0000	0.0000	0.0000	0.9992	0.0000	0.0000
6	0.0000	0.0000	0.0000	0.0000	0.0000	0.0000	0.0000
7	0.0000	0.0000	0.0000	0.0000	0.0000	0.0000	1.0000

Age 18	1	2	3	4	5	6	7
1	0.8746	0.0617	0.0355	0.0279	0.0003	0.0000	0.0000

5	0.1125	0.0000	0.0000	0.1317	0.7558	0.0000	0.0000
6	0.0000	0.0017	0.0000	0.5124	0.0000	0.4859	0.0000
7	0.0000	0.0000	0.0000	0.0000	0.0000	0.0000	1.0000

Age 9	1	2	3	4	5	6	7
1	0.8431	0.0810	0.0404	0.0350	0.0003	0.0002	0.0000
2	0.6232	0.1974	0.0914	0.0862	0.0012	0.0000	0.0006
3	0.5548	0.1035	0.2471	0.0946	0.0000	0.0000	0.0000
4	0.3588	0.0658	0.0603	0.5150	0.0000	0.0001	0.0000
5	0.1002	0.0000	0.0000	0.1384	0.7614	0.0000	0.0000
6	0.0000	0.0038	0.0000	0.5281	0.0000	0.4681	0.0000
7	0.0000	0.0000	0.0000	0.0000	0.0000	0.0000	1.0000

Age 10	1	2	3	4	5	6	7
1	0.8448	0.0804	0.0407	0.0336	0.0003	0.0002	0.0000
2	0.6253	0.1969	0.0933	0.0825	0.0011	0.0000	0.0009
3	0.5501	0.1065	0.2556	0.0878	0.0000	0.0000	0.0000
4	0.3570	0.0661	0.0643	0.5124	0.0000	0.0001	0.0001
5	0.0880	0.0000	0.0000	0.1450	0.7670	0.0000	0.0000
6	0.0000	0.0064	0.0000	0.5479	0.0000	0.4457	0.0000
7	0.0000	0.0000	0.0000	0.0000	0.0000	0.0000	1.0000

Age 11	1	2	3	4	5	6	7
1	0.8461	0.0800	0.0411	0.0323	0.0003	0.0002	0.0000
2	0.6272	0.1966	0.0952	0.0789	0.0009	0.0000	0.0012
3	0.5450	0.1095	0.2642	0.0813	0.0000	0.0000	0.0000
4	0.3551	0.0667	0.0683	0.5097	0.0000	0.0001	0.0001
5	0.0761	0.0000	0.0000	0.1515	0.7724	0.0000	0.0000
6	0.0000	0.0098	0.0000	0.5730	0.0000	0.4172	0.0000
7	0.0000	0.0000	0.0000	0.0000	0.0000	0.0000	1.0000

Age 12	1	2	3	4	5	6	7
1	0.8471	0.0798	0.0416	0.0311	0.0003	0.0001	0.0000
2	0.6286	0.1966	0.0971	0.0755	0.0008	0.0000	0.0014
3	0.5395	0.1126	0.2729	0.0750	0.0000	0.0000	0.0000
4	0.3529	0.0675	0.0724	0.5070	0.0000	0.0001	0.0001
5	0.0647	0.0000	0.0000	0.1578	0.7774	0.0000	0.0001
6	0.0000	0.0141	0.0000	0.6056	0.0000	0.3803	0.0000
7	0.0000	0.0000	0.0000	0.0000	0.0000	0.0000	1.0000

Age 13	1	2	3	4	5	6	7
1	0.8476	0.0799	0.0422	0.0299	0.0003	0.0001	0.0000
2	0.6296	0.1967	0.0990	0.0724	0.0007	0.0000	0.0016
3	0.5332	0.1159	0.2817	0.0692	0.0000	0.0000	0.0000
4	0.3504	0.0686	0.0765	0.5043	0.0000	0.0001	0.0001
5	0.0538	0.0000	0.0000	0.1640	0.7821	0.0000	0.0001
6	0.0000	0.0198	0.0000	0.6487	0.0000	0.3315	0.0000
7	0.0000	0.0000	0.0000	0.0000	0.0000	0.0000	1.0000

Age 14	1	2	3	4	5	6	7
1	0.8472	0.0803	0.0431	0.0289	0.0004	0.0001	0.0000
2	0.6300	0.1972	0.1010	0.0695	0.0005	0.0000	0.0018
3	0.5262	0.1194	0.2906	0.0638	0.0000	0.0000	0.0000
4	0.3474	0.0700	0.0807	0.5017	0.0000	0.0001	0.0001
5	0.0434	0.0000	0.0000	0.1700	0.7865	0.0000	0.0001
6	0.0000	0.0277	0.0000	0.7076	0.0000	0.2647	0.0000
7	0.0000	0.0000	0.0000	0.0000	0.0000	0.0000	1.0000

Age 15	1	2	3	4	5	6	7
1	0.8463	0.0811	0.0441	0.0280	0.0004	0.0001	0.0000
2	0.6297	0.1980	0.1029	0.0670	0.0004	0.0000	0.0020
3	0.5182	0.1232	0.2997	0.0589	0.0000	0.0000	0.0000
4	0.3441	0.0719	0.0849	0.4990	0.0000	0.0001	0.0001
5	0.0338	0.0000	0.0000	0.1758	0.7903	0.0000	0.0001
6	0.0000	0.0390	0.0000	0.7923	0.0000	0.1687	0.0000
7	0.0000	0.0000	0.0000	0.0000	0.0000	0.0000	1.0000

Age 16	1	2	3	4	5	6	7
1	0.8446	0.0823	0.0454	0.0272	0.0004	0.0001	0.0000
2	0.6285	0.1993	0.1049	0.0649	0.0003	0.0000	0.0021
3	0.5091	0.1272	0.3090	0.0547	0.0000	0.0000	0.0000
4	0.3401	0.0742	0.0891	0.4963	0.0000	0.0001	0.0001
5	0.0250	0.0000	0.0000	0.1813	0.7936	0.0000	0.0001
6	0.0000	0.0565	0.0000	0.9230	0.0000	0.0205	0.0000
7	0.0000	0.0000	0.0000	0.0000	0.0000	0.0000	1.0000

Age 17	1	2	3	4	5	6	7
1	0.8419	0.0840	0.0470	0.0266	0.0004	0.0001	0.0000
2	0.6266	0.2009	0.1070	0.0631	0.0002	0.0000	0.0022
3	0.4988	0.1315	0.3185	0.0512	0.0000	0.0000	0.0000
4	0.3356	0.0771	0.0935	0.4936	0.0000	0.0001	0.0001
5	0.0171	0.0000	0.0000	0.1865	0.7963	0.0000	0.0001
6	0.0000	0.0701	0.0000	0.9299	0.0000	0.0000	0.0000
7	0.0000	0.0000	0.0000	0.0000	0.0000	0.0000	1.0000

Age 18	1	2	3	4	5	6	7
1	0.8384	0.0862	0.0489	0.0261	0.0004	0.0000	0.0000

2	0.7250	0.1576	0.0683	0.0491	0.0000	0.0000	0.0000
3	0.5447	0.1100	0.2834	0.0578	0.0000	0.0041	0.0000
4	0.3789	0.0530	0.0330	0.5314	0.0004	0.0000	0.0033
5	0.0006	0.0000	0.0000	0.0000	0.9994	0.0000	0.0000
6	0.0000	0.0000	0.0000	0.0000	0.0000	0.0000	0.0000
7	0.0000	0.0000	0.0000	0.0000	0.0000	0.0000	1.0000

Age 19	1	2	3	4	5	6	7
1	0.8748	0.0614	0.0362	0.0273	0.0003	0.0000	0.0000
2	0.7259	0.1567	0.0689	0.0485	0.0000	0.0000	0.0000
3	0.5407	0.1119	0.2872	0.0561	0.0000	0.0041	0.0000
4	0.3733	0.0515	0.0334	0.5383	0.0002	0.0000	0.0033
5	0.0004	0.0000	0.0000	0.0000	0.9996	0.0000	0.0000
6	0.0000	0.0000	0.0000	0.0000	0.0000	0.0000	0.0000
7	0.0000	0.0000	0.0000	0.0000	0.0000	0.0000	1.0000

Age 20	1	2	3	4	5	6	7
1	0.8740	0.0615	0.0372	0.0270	0.0003	0.0000	0.0000
2	0.7251	0.1563	0.0701	0.0485	0.0000	0.0000	0.0000
3	0.5355	0.1142	0.2913	0.0551	0.0000	0.0039	0.0000
4	0.3648	0.0503	0.0346	0.5472	0.0000	0.0000	0.0031
5	0.0000	0.0000	0.0000	0.0000	1.0000	0.0000	0.0000
6	0.0000	0.0000	0.0000	0.0000	0.0000	0.0000	0.0000
7	0.0000	0.0000	0.0000	0.0000	0.0000	0.0000	1.0000

Age 21	1	2	3	4	5	6	7
1	0.8723	0.0622	0.0383	0.0269	0.0003	0.0000	0.0000
2	0.7224	0.1566	0.0718	0.0492	0.0000	0.0000	0.0000
3	0.5289	0.1167	0.2958	0.0547	0.0002	0.0037	0.0000
4	0.3529	0.0494	0.0364	0.5581	0.0000	0.0003	0.0029
5	0.0000	0.0000	0.0002	0.0116	0.9879	0.0000	0.0003
6	0.0000	0.0000	0.0000	0.0000	0.0000	0.0000	0.0000
7	0.0000	0.0000	0.0000	0.0000	0.0000	0.0000	1.0000

Age 22	1	2	3	4	5	6	7
1	0.8696	0.0633	0.0397	0.0270	0.0002	0.0001	0.0001
2	0.7178	0.1575	0.0741	0.0506	0.0000	0.0000	0.0000
3	0.5211	0.1193	0.3007	0.0549	0.0006	0.0034	0.0000
4	0.3384	0.0487	0.0389	0.5708	0.0000	0.0006	0.0026
5	0.0000	0.0000	0.0005	0.0249	0.9740	0.0000	0.0006
6	0.0000	0.0000	0.0000	0.0000	0.0000	0.0000	0.0000
7	0.0000	0.0000	0.0000	0.0000	0.0000	0.0000	1.0000

Age 23	1	2	3	4	5	6	7
1	0.8664	0.0647	0.0411	0.0274	0.0002	0.0001	0.0001
2	0.7120	0.1588	0.0768	0.0523	0.0000	0.0001	0.0000
3	0.5125	0.1219	0.3059	0.0557	0.0009	0.0030	0.0001
4	0.3220	0.0482	0.0420	0.5847	0.0000	0.0009	0.0022
5	0.0000	0.0000	0.0007	0.0395	0.9589	0.0000	0.0009
6	0.0000	0.0000	0.0000	0.0000	0.0000	0.0000	0.0000
7	0.0000	0.0000	0.0000	0.0000	0.0000	0.0000	1.0000

Age 24	1	2	3	4	5	6	7
1	0.8626	0.0664	0.0427	0.0279	0.0002	0.0001	0.0001
2	0.7050	0.1606	0.0799	0.0544	0.0000	0.0001	0.0000
3	0.5034	0.1243	0.3115	0.0569	0.0013	0.0025	0.0001
4	0.3042	0.0480	0.0454	0.5993	0.0000	0.0013	0.0018
5	0.0000	0.0000	0.0009	0.0547	0.9432	0.0000	0.0012
6	0.0000	0.0000	0.0000	0.0000	0.0000	0.0000	0.0000
7	0.0000	0.0000	0.0000	0.0000	0.0000	0.0000	1.0000

Age 25	1	2	3	4	5	6	7
1	0.8584	0.0683	0.0443	0.0285	0.0001	0.0002	0.0002
2	0.6971	0.1627	0.0833	0.0568	0.0000	0.0001	0.0000
3	0.4939	0.1262	0.3175	0.0586	0.0017	0.0020	0.0001
4	0.2859	0.0479	0.0491	0.6141	0.0000	0.0016	0.0014
5	0.0000	0.0000	0.0011	0.0699	0.9276	0.0000	0.0014
6	0.3333	0.0000	0.3333	0.0000	0.0000	0.3333	0.0000
7	0.0000	0.0000	0.0000	0.0000	0.0000	0.0000	1.0000

Age 26	1	2	3	4	5	6	7
1	0.8540	0.0703	0.0459	0.0293	0.0001	0.0002	0.0002
2	0.6887	0.1652	0.0869	0.0591	0.0000	0.0001	0.0000
3	0.4844	0.1276	0.3237	0.0606	0.0020	0.0016	0.0001
4	0.2676	0.0480	0.0531	0.6284	0.0000	0.0019	0.0010
5	0.0000	0.0000	0.0011	0.0844	0.9130	0.0000	0.0015
6	0.3333	0.0000	0.3333	0.0000	0.0000	0.3333	0.0000
7	0.0000	0.0000	0.0000	0.0000	0.0000	0.0000	1.0000

Age 27	1	2	3	4	5	6	7
1	0.8495	0.0724	0.0475	0.0301	0.0001	0.0002	0.0002
2	0.6797	0.1679	0.0908	0.0615	0.0000	0.0001	0.0000
3	0.4750	0.1282	0.3303	0.0630	0.0023	0.0011	0.0001
4	0.2500	0.0481	0.0571	0.6419	0.0000	0.0022	0.0007
5	0.0000	0.0000	0.0011	0.0978	0.8997	0.0000	0.0014
6	0.3333	0.0000	0.3333	0.0000	0.0000	0.3333	0.0000
7	0.0000	0.0000	0.0000	0.0000	0.0000	0.0000	1.0000

2	0.6236	0.2031	0.1090	0.0619	0.0001	0.0000	0.0023
3	0.4872	0.1361	0.3282	0.0484	0.0000	0.0000	0.0000
4	0.3305	0.0806	0.0979	0.4909	0.0000	0.0001	0.0001
5	0.0103	0.0000	0.0000	0.1914	0.7983	0.0000	0.0000
6	0.0000	0.0847	0.0000	0.9153	0.0000	0.0000	0.0000
7	0.0000	0.0000	0.0000	0.0000	0.0000	0.0000	1.0000

Age 19	1	2	3	4	5	6	7
1	0.8337	0.0890	0.0511	0.0258	0.0004	0.0000	0.0000
2	0.6195	0.2058	0.1111	0.0612	0.0001	0.0000	0.0023
3	0.4742	0.1411	0.3382	0.0465	0.0000	0.0000	0.0000
4	0.3246	0.0847	0.1024	0.4882	0.0000	0.0000	0.0000
5	0.0045	0.0000	0.0000	0.1959	0.7996	0.0000	0.0000
6	0.0000	0.1020	0.0000	0.8980	0.0000	0.0000	0.0000
7	0.0000	0.0000	0.0000	0.0000	0.0000	0.0000	1.0000

Age 20	1	2	3	4	5	6	7
1	0.8276	0.0925	0.0537	0.0258	0.0004	0.0000	0.0000
2	0.6143	0.2092	0.1133	0.0610	0.0000	0.0000	0.0022
3	0.4596	0.1465	0.3485	0.0455	0.0000	0.0000	0.0000
4	0.3179	0.0896	0.1069	0.4855	0.0000	0.0000	0.0000
5	0.0000	0.0000	0.0000	0.2000	0.8000	0.0000	0.0000
6	0.0000	0.0000	0.0000	0.0000	0.0000	0.0000	0.0000
7	0.0000	0.0000	0.0000	0.0000	0.0000	0.0000	1.0000

Age 21	1	2	3	4	5	6	7
1	0.8206	0.0966	0.0566	0.0259	0.0003	0.0000	0.0000
2	0.6080	0.2130	0.1155	0.0614	0.0000	0.0001	0.0020
3	0.4433	0.1522	0.3590	0.0454	0.0000	0.0000	0.0000
4	0.3103	0.0951	0.1116	0.4828	0.0002	0.0000	0.0000
5	0.0000	0.0000	0.0001	0.2030	0.7969	0.0000	0.0000
6	0.5944	0.0000	0.0012	0.0000	0.0000	0.4024	0.0020
7	0.0000	0.0000	0.0000	0.0000	0.0000	0.0000	1.0000

Age 22	1	2	3	4	5	6	7
1	0.8121	0.1013	0.0599	0.0263	0.0003	0.0000	0.0001
2	0.6003	0.2174	0.1177	0.0624	0.0000	0.0003	0.0019
3	0.4258	0.1582	0.3698	0.0462	0.0000	0.0000	0.0000
4	0.3020	0.1011	0.1162	0.4802	0.0005	0.0000	0.0000
5	0.0000	0.0000	0.0003	0.2056	0.7941	0.0000	0.0000
6	0.5725	0.0000	0.0011	0.0000	0.0000	0.4245	0.0019
7	0.0000	0.0000	0.0000	0.0000	0.0000	0.0000	1.0000

Age 23	1	2	3	4	5	6	7
1	0.8032	0.1062	0.0634	0.0268	0.0003	0.0000	0.0001
2	0.5923	0.2220	0.1200	0.0637	0.0000	0.0004	0.0016
3	0.4075	0.1640	0.3807	0.0478	0.0000	0.0000	0.0000
4	0.2930	0.1072	0.1209	0.4782	0.0007	0.0000	0.0000
5	0.0000	0.0000	0.0005	0.2079	0.7916	0.0000	0.0000
6	0.5564	0.0000	0.0010	0.0000	0.0000	0.4408	0.0018
7	0.0000	0.0000	0.0000	0.0000	0.0000	0.0000	1.0000

Age 24	1	2	3	4	5	6	7
1	0.7941	0.1111	0.0671	0.0274	0.0002	0.0000	0.0001
2	0.5838	0.2265	0.1224	0.0653	0.0000	0.0006	0.0014
3	0.3888	0.1695	0.3918	0.0499	0.0000	0.0000	0.0000
4	0.2835	0.1130	0.1255	0.4770	0.0010	0.0000	0.0000
5	0.0000	0.0000	0.0006	0.2095	0.7899	0.0000	0.0000
6	0.5440	0.0000	0.0009	0.0000	0.0000	0.4535	0.0016
7	0.0000	0.0000	0.0000	0.0000	0.0000	0.0000	1.0000

Age 25	1	2	3	4	5	6	7
1	0.7845	0.1160	0.0709	0.0282	0.0002	0.0000	0.0002
2	0.5751	0.2309	0.1249	0.0672	0.0000	0.0008	0.0011
3	0.3702	0.1743	0.4029	0.0525	0.0000	0.0000	0.0000
4	0.2737	0.1183	0.1299	0.4768	0.0013	0.0000	0.0000
5	0.0000	0.0000	0.0007	0.2105	0.7888	0.0000	0.0000
6	0.5341	0.0000	0.0008	0.0000	0.0000	0.4637	0.0014
7	0.0000	0.0000	0.0000	0.0000	0.0000	0.0000	1.0000

Age 26	1	2	3	4	5	6	7
1	0.7756	0.1205	0.0746	0.0290	0.0001	0.0000	0.0002
2	0.5666	0.2349	0.1276	0.0691	0.0000	0.0009	0.0009
3	0.3522	0.1783	0.4140	0.0554	0.0000	0.0000	0.0000
4	0.2636	0.1227	0.1341	0.4779	0.0016	0.0000	0.0000
5	0.0000	0.0000	0.0007	0.2106	0.7887	0.0000	0.0000
6	0.5256	0.0000	0.0007	0.0000	0.0000	0.4724	0.0013
7	0.0000	0.0000	0.0000	0.0000	0.0000	0.0000	1.0000

Age 27	1	2	3	4	5	6	7
1	0.7671	0.1245	0.0782	0.0299	0.0001	0.0000	0.0002
2	0.5587	0.2383	0.1303	0.0711	0.0000	0.0010	0.0006
3	0.3352	0.1811	0.4251	0.0585	0.0000	0.0000	0.0000
4	0.2535	0.1259	0.1382	0.4806	0.0018	0.0000	0.0000
5	0.0000	0.0000	0.0007	0.2097	0.7896	0.0000	0.0000
6	0.5184	0.0000	0.0006	0.0000	0.0000	0.4800	0.0010
7	0.0000	0.0000	0.0000	0.0000	0.0000	0.0000	1.0000

Age 28	1	2	3	4	5	6	7
1	0.8451	0.0744	0.0490	0.0310	0.0001	0.0002	0.0002
2	0.6709	0.1707	0.0947	0.0636	0.0000	0.0001	0.0000
3	0.4661	0.1279	0.3371	0.0656	0.0026	0.0007	0.0001
4	0.2339	0.0484	0.0611	0.6538	0.0000	0.0024	0.0004
5	0.0000	0.0000	0.0009	0.1094	0.8885	0.0000	0.0012
6	0.3333	0.0000	0.3333	0.0000	0.0000	0.3333	0.0000
7	0.0000	0.0000	0.0000	0.0000	0.0000	0.0000	1.0000

Age 29	1	2	3	4	5	6	7
1	0.8410	0.0762	0.0504	0.0318	0.0000	0.0003	0.0003
2	0.6621	0.1737	0.0987	0.0654	0.0000	0.0001	0.0000
3	0.4578	0.1264	0.3443	0.0684	0.0027	0.0003	0.0000
4	0.2197	0.0487	0.0650	0.6640	0.0000	0.0025	0.0001
5	0.0000	0.0000	0.0006	0.1186	0.8801	0.0000	0.0007
6	0.3333	0.0000	0.3333	0.0000	0.0000	0.3333	0.0000
7	0.0000	0.0000	0.0000	0.0000	0.0000	0.0000	1.0000

Age 30	1	2	3	4	5	6	7
1	0.8373	0.0778	0.0516	0.0327	0.0000	0.0003	0.0003
2	0.6540	0.1766	0.1026	0.0668	0.0000	0.0000	0.0000
3	0.4506	0.1236	0.3517	0.0714	0.0027	0.0000	0.0000
4	0.2083	0.0490	0.0686	0.6716	0.0000	0.0025	0.0000
5	0.0000	0.0000	0.0000	0.1250	0.8750	0.0000	0.0000
6	0.3333	0.0000	0.3333	0.0000	0.0000	0.3333	0.0000
7	0.0000	0.0000	0.0000	0.0000	0.0000	0.0000	1.0000

Age 31	1	2	3	4	5	6	7
1	0.8342	0.0791	0.0526	0.0335	0.0000	0.0003	0.0003
2	0.6463	0.1795	0.1065	0.0677	0.0000	0.0000	0.0000
3	0.4442	0.1194	0.3592	0.0745	0.0027	0.0000	0.0000
4	0.2000	0.0493	0.0719	0.6758	0.0007	0.0023	0.0000
5	0.0016	0.0000	0.0000	0.1276	0.8707	0.0001	0.0000
6	0.3333	0.0000	0.3333	0.0000	0.0000	0.3333	0.0000
7	0.0000	0.0000	0.0000	0.0000	0.0000	0.0000	1.0000

Age 32	1	2	3	4	5	6	7
1	0.8316	0.0801	0.0534	0.0343	0.0000	0.0003	0.0003
2	0.6394	0.1823	0.1101	0.0682	0.0000	0.0000	0.0000
3	0.4388	0.1140	0.3669	0.0778	0.0025	0.0000	0.0000
4	0.1944	0.0496	0.0748	0.6776	0.0015	0.0020	0.0001
5	0.0036	0.0000	0.0000	0.1269	0.8694	0.0001	0.0000
6	0.3333	0.0000	0.3333	0.0000	0.0000	0.3333	0.0000
7	0.0000	0.0000	0.0000	0.0000	0.0000	0.0000	1.0000

Age 33	1	2	3	4	5	6	7
1	0.8295	0.0808	0.0540	0.0351	0.0000	0.0003	0.0003
2	0.6330	0.1850	0.1135	0.0685	0.0000	0.0000	0.0000
3	0.4340	0.1079	0.3747	0.0812	0.0022	0.0000	0.0000
4	0.1910	0.0497	0.0773	0.6775	0.0025	0.0017	0.0003
5	0.0060	0.0000	0.0000	0.1235	0.8703	0.0002	0.0000
6	0.3333	0.0000	0.3333	0.0000	0.0000	0.3333	0.0000
7	0.0000	0.0000	0.0000	0.0000	0.0000	0.0000	1.0000

Age 34	1	2	3	4	5	6	7
1	0.8276	0.0812	0.0546	0.0360	0.0000	0.0003	0.0003
2	0.6269	0.1875	0.1166	0.0690	0.0000	0.0000	0.0000
3	0.4295	0.1013	0.3825	0.0849	0.0018	0.0000	0.0000
4	0.1892	0.0497	0.0793	0.6765	0.0035	0.0013	0.0005
5	0.0088	0.0000	0.0000	0.1179	0.8730	0.0003	0.0000
6	0.3333	0.0000	0.3333	0.0000	0.0000	0.3333	0.0000
7	0.0000	0.0000	0.0000	0.0000	0.0000	0.0000	1.0000

Age 35	1	2	3	4	5	6	7
1	0.8261	0.0814	0.0551	0.0370	0.0000	0.0002	0.0002
2	0.6214	0.1897	0.1192	0.0697	0.0000	0.0000	0.0000
3	0.4248	0.0946	0.3903	0.0889	0.0014	0.0000	0.0000
4	0.1885	0.0496	0.0809	0.6748	0.0045	0.0009	0.0008
5	0.0119	0.0000	0.0000	0.1104	0.8774	0.0003	0.0000
6	0.1667	0.0000	0.0833	0.0833	0.0000	0.5834	0.0833
7	0.0000	0.0000	0.0000	0.0000	0.0000	0.0000	1.0000

Age 36	1	2	3	4	5	6	7
1	0.8244	0.0814	0.0556	0.0382	0.0000	0.0002	0.0002
2	0.6160	0.1917	0.1214	0.0709	0.0000	0.0000	0.0000
3	0.4197	0.0881	0.3978	0.0933	0.0010	0.0000	0.0000
4	0.1882	0.0492	0.0821	0.6735	0.0054	0.0005	0.0011
5	0.0153	0.0000	0.0000	0.1016	0.8828	0.0003	0.0000
6	0.1667	0.0000	0.0833	0.0833	0.0000	0.5834	0.0833
7	0.0000	0.0000	0.0000	0.0000	0.0000	0.0000	1.0000

Age 37	1	2	3	4	5	6	7
1	0.8227	0.0812	0.0561	0.0396	0.0000	0.0002	0.0002
2	0.6110	0.1934	0.1229	0.0727	0.0000	0.0000	0.0000
3	0.4139	0.0822	0.4051	0.0981	0.0006	0.0000	0.0000
4	0.1877	0.0486	0.0828	0.6730	0.0063	0.0002	0.0014

Age 28	1	2	3	4	5	6	7
1	0.7593	0.1278	0.0817	0.0308	0.0001	0.0000	0.0003
2	0.5515	0.2408	0.1333	0.0729	0.0000	0.0011	0.0004
3	0.3197	0.1825	0.4360	0.0617	0.0000	0.0000	0.0000
4	0.2433	0.1275	0.1419	0.4853	0.0021	0.0000	0.0000
5	0.0000	0.0000	0.0006	0.2078	0.7916	0.0000	0.0000
6	0.5119	0.0000	0.0004	0.0000	0.0000	0.4869	0.0008
7	0.0000	0.0000	0.0000	0.0000	0.0000	0.0000	1.0000

Age 29	1	2	3	4	5	6	7
1	0.7531	0.1301	0.0848	0.0317	0.0000	0.0000	0.0003
2	0.5454	0.2423	0.1364	0.0745	0.0000	0.0012	0.0002
3	0.3062	0.1823	0.4467	0.0648	0.0000	0.0000	0.0000
4	0.2333	0.1272	0.1453	0.4920	0.0022	0.0000	0.0000
5	0.0000	0.0000	0.0004	0.2046	0.7950	0.0000	0.0000
6	0.5060	0.0000	0.0002	0.0000	0.0000	0.4934	0.0004
7	0.0000	0.0000	0.0000	0.0000	0.0000	0.0000	1.0000

Age 30	1	2	3	4	5	6	7
1	0.7482	0.1312	0.0877	0.0326	0.0000	0.0000	0.0003
2	0.5409	0.2426	0.1396	0.0757	0.0000	0.0012	0.0000
3	0.2950	0.1801	0.4572	0.0677	0.0000	0.0000	0.0000
4	0.2235	0.1247	0.1482	0.5012	0.0024	0.0000	0.0000
5	0.0000	0.0000	0.0000	0.2000	0.8000	0.0000	0.0000
6	0.5000	0.0000	0.0000	0.0000	0.0000	0.5000	0.0000
7	0.0000	0.0000	0.0000	0.0000	0.0000	0.0000	1.0000

Age 31	1	2	3	4	5	6	7
1	0.7449	0.1310	0.0901	0.0335	0.0000	0.0001	0.0004
2	0.5377	0.2415	0.1431	0.0766	0.0000	0.0011	0.0000
3	0.2865	0.1758	0.4674	0.0703	0.0001	0.0000	0.0000
4	0.2141	0.1197	0.1507	0.5128	0.0024	0.0002	0.0001
5	0.0010	0.0000	0.0000	0.1936	0.8053	0.0000	0.0001
6	0.4768	0.0102	0.0000	0.0233	0.0000	0.4896	0.0000
7	0.0000	0.0000	0.0000	0.0000	0.0000	0.0000	1.0000

Age 32	1	2	3	4	5	6	7
1	0.7433	0.1297	0.0922	0.0342	0.0000	0.0002	0.0004
2	0.5358	0.2391	0.1468	0.0772	0.0000	0.0010	0.0000
3	0.2804	0.1697	0.4772	0.0726	0.0001	0.0000	0.0000
4	0.2049	0.1127	0.1527	0.5266	0.0024	0.0004	0.0003
5	0.0017	0.0000	0.0000	0.1861	0.8120	0.0000	0.0002
6	0.4516	0.0220	0.0000	0.0483	0.0000	0.4781	0.0000
7	0.0000	0.0000	0.0000	0.0000	0.0000	0.0000	1.0000

Age 33	1	2	3	4	5	6	7
1	0.7430	0.1274	0.0939	0.0350	0.0000	0.0003	0.0004
2	0.5347	0.2358	0.1508	0.0778	0.0000	0.0009	0.0000
3	0.2762	0.1621	0.4867	0.0748	0.0002	0.0000	0.0000
4	0.1961	0.1041	0.1541	0.5421	0.0024	0.0007	0.0005
5	0.0022	0.0000	0.0000	0.1777	0.8198	0.0000	0.0003
6	0.4241	0.0349	0.0000	0.0748	0.0000	0.4662	0.0000
7	0.0000	0.0000	0.0000	0.0000	0.0000	0.0000	1.0000

Age 34	1	2	3	4	5	6	7
1	0.7439	0.1243	0.0953	0.0357	0.0000	0.0004	0.0004
2	0.5339	0.2319	0.1551	0.0783	0.0000	0.0008	0.0000
3	0.2733	0.1534	0.4959	0.0770	0.0003	0.0000	0.0000
4	0.1875	0.0944	0.1550	0.5591	0.0023	0.0010	0.0007
5	0.0023	0.0000	0.0000	0.1687	0.8286	0.0000	0.0004
6	0.3943	0.0487	0.0000	0.1023	0.0000	0.4547	0.0000
7	0.0000	0.0000	0.0000	0.0000	0.0000	0.0000	1.0000

Age 35	1	2	3	4	5	6	7
1	0.7457	0.1207	0.0964	0.0363	0.0000	0.0005	0.0004
2	0.5332	0.2274	0.1597	0.0791	0.0000	0.0006	0.0000
3	0.2714	0.1440	0.5049	0.0793	0.0004	0.0000	0.0000
4	0.1792	0.0841	0.1554	0.5768	0.0022	0.0013	0.0010
5	0.0023	0.0000	0.0000	0.1594	0.8379	0.0000	0.0004
6	0.3621	0.0631	0.0000	0.1303	0.0000	0.4445	0.0000
7	0.0000	0.0000	0.0000	0.0000	0.0000	0.0000	1.0000

Age 36	1	2	3	4	5	6	7
1	0.7480	0.1167	0.0973	0.0370	0.0000	0.0006	0.0004
2	0.5319	0.2227	0.1647	0.0803	0.0000	0.0004	0.0000
3	0.2698	0.1343	0.5136	0.0818	0.0005	0.0000	0.0000
4	0.1711	0.0737	0.1551	0.5953	0.0021	0.0015	0.0012
5	0.0020	0.0000	0.0000	0.1501	0.8475	0.0000	0.0004
6	0.3279	0.0774	0.0000	0.1579	0.0000	0.4368	0.0000
7	0.0000	0.0000	0.0000	0.0000	0.0000	0.0000	1.0000

Age 37	1	2	3	4	5	6	7
1	0.7506	0.1125	0.0981	0.0377	0.0000	0.0007	0.0004
2	0.5295	0.2181	0.1701	0.0820	0.0000	0.0003	0.0000
3	0.2682	0.1246	0.5219	0.0847	0.0006	0.0000	0.0000
4	0.1631	0.0637	0.1543	0.6137	0.0020	0.0017	0.0015

5	0.0189	0.0000	0.0000	0.0919	0.8889	0.0003	0.0000
6	0.1667	0.0000	0.0833	0.0833	0.0000	0.5834	0.0833
7	0.0000	0.0000	0.0000	0.0000	0.0000	0.0000	1.0000

Age 38	1	2	3	4	5	6	7
1	0.8210	0.0807	0.0567	0.0412	0.0000	0.0002	0.0002
2	0.6058	0.1948	0.1238	0.0756	0.0000	0.0000	0.0000
3	0.4071	0.0773	0.4120	0.1034	0.0003	0.0000	0.0000
4	0.1865	0.0477	0.0829	0.6741	0.0070	0.0001	0.0017
5	0.0229	0.0000	0.0000	0.0818	0.8950	0.0003	0.0000
6	0.1667	0.0000	0.0833	0.0833	0.0000	0.5834	0.0833
7	0.0000	0.0000	0.0000	0.0000	0.0000	0.0000	1.0000

Age 39	1	2	3	4	5	6	7
1	0.8189	0.0801	0.0574	0.0432	0.0000	0.0002	0.0002
2	0.6009	0.1957	0.1239	0.0795	0.0000	0.0000	0.0000
3	0.3988	0.0736	0.4184	0.1092	0.0000	0.0000	0.0000
4	0.1841	0.0465	0.0825	0.6774	0.0076	0.0000	0.0019
5	0.0270	0.0000	0.0000	0.0718	0.9010	0.0002	0.0000
6	0.1667	0.0000	0.0833	0.0833	0.0000	0.5834	0.0833
7	0.0000	0.0000	0.0000	0.0000	0.0000	0.0000	1.0000

Age 40	1	2	3	4	5	6	7
1	0.8166	0.0793	0.0582	0.0455	0.0000	0.0002	0.0002
2	0.5957	0.1962	0.1231	0.0847	0.0000	0.0003	0.0000
3	0.3887	0.0714	0.4241	0.1155	0.0000	0.0000	0.0002
4	0.1798	0.0449	0.0816	0.6836	0.0079	0.0001	0.0021
5	0.0313	0.0000	0.0000	0.0625	0.9062	0.0000	0.0000
6	0.1667	0.0000	0.0833	0.0833	0.0000	0.5834	0.0833
7	0.0000	0.0000	0.0000	0.0000	0.0000	0.0000	1.0000

Age 41	1	2	3	4	5	6	7
1	0.8141	0.0783	0.0591	0.0480	0.0001	0.0002	0.0002
2	0.5904	0.1962	0.1217	0.0911	0.0000	0.0006	0.0000
3	0.3772	0.0706	0.4294	0.1222	0.0000	0.0000	0.0005
4	0.1740	0.0430	0.0802	0.6922	0.0080	0.0003	0.0022
5	0.0354	0.0000	0.0029	0.0539	0.9041	0.0000	0.0037
6	0.1667	0.0000	0.0833	0.0833	0.0000	0.5834	0.0833
7	0.0000	0.0000	0.0000	0.0000	0.0000	0.0000	1.0000

Age 42	1	2	3	4	5	6	7
1	0.8111	0.0773	0.0601	0.0509	0.0002	0.0002	0.0002
2	0.5848	0.1958	0.1199	0.0985	0.0000	0.0010	0.0000
3	0.3647	0.0709	0.4343	0.1292	0.0000	0.0000	0.0008
4	0.1668	0.0409	0.0784	0.7028	0.0080	0.0007	0.0024
5	0.0394	0.0000	0.0064	0.0467	0.8992	0.0000	0.0083
6	0.1667	0.0000	0.0833	0.0833	0.0000	0.5834	0.0833
7	0.0000	0.0000	0.0000	0.0000	0.0000	0.0000	1.0000

Age 43	1	2	3	4	5	6	7
1	0.8081	0.0761	0.0612	0.0539	0.0002	0.0002	0.0003
2	0.5790	0.1950	0.1178	0.1067	0.0000	0.0015	0.0000
3	0.3515	0.0720	0.4390	0.1364	0.0000	0.0000	0.0011
4	0.1586	0.0386	0.0763	0.7151	0.0079	0.0011	0.0024
5	0.0432	0.0000	0.0103	0.0413	0.8918	0.0000	0.0134
6	0.1667	0.0000	0.0833	0.0833	0.0000	0.5834	0.0833
7	0.0000	0.0000	0.0000	0.0000	0.0000	0.0000	1.0000

Age 44	1	2	3	4	5	6	7
1	0.8047	0.0750	0.0623	0.0572	0.0003	0.0002	0.0003
2	0.5731	0.1938	0.1156	0.1156	0.0000	0.0019	0.0000
3	0.3379	0.0737	0.4434	0.1436	0.0000	0.0000	0.0014
4	0.1496	0.0361	0.0738	0.7288	0.0076	0.0016	0.0025
5	0.0467	0.0000	0.0143	0.0377	0.8825	0.0000	0.0188
6	0.1667	0.0000	0.0833	0.0833	0.0000	0.5834	0.0833
7	0.0000	0.0000	0.0000	0.0000	0.0000	0.0000	1.0000

Age 45	1	2	3	4	5	6	7
1	0.8013	0.0737	0.0634	0.0606	0.0004	0.0002	0.0004
2	0.5668	0.1923	0.1136	0.1249	0.0000	0.0024	0.0000
3	0.3242	0.0756	0.4477	0.1507	0.0000	0.0000	0.0017
4	0.1401	0.0335	0.0712	0.7431	0.0074	0.0021	0.0026
5	0.0498	0.0000	0.0182	0.0364	0.8713	0.0000	0.0243
6	0.1500	0.0000	0.0500	0.0500	0.0000	0.6500	0.1000
7	0.0000	0.0000	0.0000	0.0000	0.0000	0.0000	1.0000

Age 46	1	2	3	4	5	6	7
1	0.7974	0.0726	0.0646	0.0641	0.0005	0.0002	0.0006
2	0.5602	0.1904	0.1119	0.1346	0.0000	0.0029	0.0000
3	0.3108	0.0776	0.4520	0.1576	0.0000	0.0000	0.0020
4	0.1303	0.0310	0.0684	0.7579	0.0071	0.0026	0.0027
5	0.0525	0.0000	0.0218	0.0373	0.8587	0.0000	0.0297
6	0.1500	0.0000	0.0500	0.0500	0.0000	0.6500	0.1000
7	0.0000	0.0000	0.0000	0.0000	0.0000	0.0000	1.0000

Age 47	1	2	3	4	5	6	7
1	0.7936	0.0714	0.0657	0.0677	0.0006	0.0003	0.0007

5	0.0015	0.0000	0.0000	0.1411	0.8570	0.0000	0.0004
6	0.2924	0.0909	0.0000	0.1837	0.0000	0.4331	0.0000
7	0.0000	0.0000	0.0000	0.0000	0.0000	0.0000	1.0000

Age 38	1	2	3	4	5	6	7
1	0.7536	0.1083	0.0987	0.0383	0.0000	0.0008	0.0003
2	0.5258	0.2138	0.1759	0.0844	0.0000	0.0001	0.0000
3	0.2661	0.1153	0.5300	0.0879	0.0007	0.0000	0.0000
4	0.1553	0.0546	0.1527	0.6318	0.0020	0.0019	0.0017
5	0.0008	0.0000	0.0000	0.1326	0.8664	0.0000	0.0002
6	0.2566	0.1025	0.0000	0.2059	0.0000	0.4350	0.0000
7	0.0000	0.0000	0.0000	0.0000	0.0000	0.0000	1.0000

Age 39	1	2	3	4	5	6	7
1	0.7563	0.1044	0.0992	0.0390	0.0000	0.0008	0.0003
2	0.5202	0.2099	0.1822	0.0877	0.0000	0.0000	0.0000
3	0.2629	0.1067	0.5380	0.0917	0.0007	0.0000	0.0000
4	0.1476	0.0469	0.1505	0.6490	0.0020	0.0020	0.0020
5	0.0000	0.0000	0.0000	0.1250	0.8750	0.0000	0.0000
6	0.2222	0.1111	0.0000	0.2222	0.0000	0.4444	0.0000
7	0.0000	0.0000	0.0000	0.0000	0.0000	0.0000	1.0000

Age 40	1	2	3	4	5	6	7
1	0.7587	0.1008	0.0997	0.0398	0.0000	0.0008	0.0002
2	0.5123	0.2068	0.1890	0.0919	0.0000	0.0000	0.0000
3	0.2582	0.0992	0.5455	0.0961	0.0008	0.0001	0.0001
4	0.1399	0.0409	0.1477	0.6653	0.0020	0.0020	0.0022
5	0.0000	0.0000	0.0017	0.1181	0.8801	0.0001	0.0000
6	0.1906	0.1150	0.0012	0.2298	0.0000	0.4614	0.0020
7	0.0000	0.0000	0.0000	0.0000	0.0000	0.0000	1.0000

Age 41	1	2	3	4	5	6	7
1	0.7607	0.0976	0.1000	0.0406	0.0001	0.0009	0.0001
2	0.5027	0.2044	0.1960	0.0969	0.0000	0.0000	0.0000
3	0.2523	0.0927	0.5529	0.1010	0.0008	0.0002	0.0001
4	0.1323	0.0365	0.1443	0.6804	0.0021	0.0019	0.0025
5	0.0000	0.0000	0.0039	0.1126	0.8834	0.0001	0.0000
6	0.1629	0.1146	0.0028	0.2295	0.0000	0.4855	0.0047
7	0.0000	0.0000	0.0000	0.0000	0.0000	0.0000	1.0000

Age 42	1	2	3	4	5	6	7
1	0.7626	0.0947	0.1003	0.0415	0.0001	0.0008	0.0000
2	0.4917	0.2026	0.2030	0.1027	0.0000	0.0000	0.0000
3	0.2453	0.0871	0.5599	0.1064	0.0008	0.0003	0.0002
4	0.1249	0.0336	0.1404	0.6943	0.0023	0.0018	0.0027
5	0.0000	0.0000	0.0064	0.1085	0.8849	0.0002	0.0000
6	0.1394	0.1105	0.0049	0.2230	0.0000	0.5143	0.0079
7	0.0000	0.0000	0.0000	0.0000	0.0000	0.0000	1.0000

Age 43	1	2	3	4	5	6	7
1	0.7638	0.0921	0.1005	0.0427	0.0001	0.0008	0.0000
2	0.4798	0.2014	0.2099	0.1089	0.0000	0.0000	0.0000
3	0.2374	0.0823	0.5667	0.1121	0.0008	0.0004	0.0003
4	0.1177	0.0317	0.1362	0.7073	0.0025	0.0017	0.0029
5	0.0000	0.0000	0.0091	0.1063	0.8843	0.0003	0.0000
6	0.1200	0.1032	0.0076	0.2118	0.0000	0.5459	0.0115
7	0.0000	0.0000	0.0000	0.0000	0.0000	0.0000	1.0000

Age 44	1	2	3	4	5	6	7
1	0.7645	0.0897	0.1006	0.0442	0.0002	0.0008	0.0000
2	0.4674	0.2007	0.2164	0.1156	0.0000	0.0000	0.0000
3	0.2288	0.0782	0.5732	0.1181	0.0008	0.0005	0.0004
4	0.1107	0.0308	0.1319	0.7193	0.0027	0.0016	0.0030
5	0.0000	0.0000	0.0120	0.1060	0.8817	0.0003	0.0000
6	0.1047	0.0936	0.0109	0.1979	0.0000	0.5775	0.0154
7	0.0000	0.0000	0.0000	0.0000	0.0000	0.0000	1.0000

Age 45	1	2	3	4	5	6	7
1	0.7648	0.0876	0.1006	0.0461	0.0002	0.0007	0.0000
2	0.4549	0.2005	0.2222	0.1224	0.0000	0.0000	0.0000
3	0.2199	0.0747	0.5792	0.1242	0.0008	0.0007	0.0005
4	0.1040	0.0305	0.1274	0.7304	0.0030	0.0015	0.0032
5	0.0000	0.0000	0.0148	0.1079	0.8770	0.0003	0.0000
6	0.0930	0.0825	0.0147	0.1831	0.0000	0.6071	0.0196
7	0.0000	0.0000	0.0000	0.0000	0.0000	0.0000	1.0000

Age 46	1	2	3	4	5	6	7
1	0.7646	0.0856	0.1005	0.0484	0.0003	0.0006	0.0000
2	0.4429	0.2006	0.2272	0.1293	0.0000	0.0000	0.0000
3	0.2108	0.0717	0.5849	0.1304	0.0008	0.0008	0.0006
4	0.0975	0.0306	0.1231	0.7408	0.0033	0.0014	0.0033
5	0.0000	0.0000	0.0175	0.1123	0.8699	0.0003	0.0000
6	0.0846	0.0706	0.0192	0.1690	0.0000	0.6327	0.0239
7	0.0000	0.0000	0.0000	0.0000	0.0000	0.0000	1.0000

Age 47	1	2	3	4	5	6	7
1	0.7640	0.0837	0.1002	0.0512	0.0003	0.0006	0.0000

2	0.5532	0.1883	0.1107	0.1444	0.0000	0.0034	0.0000
3	0.2981	0.0793	0.4563	0.1641	0.0000	0.0000	0.0022
4	0.1206	0.0284	0.0655	0.7726	0.0069	0.0031	0.0029
5	0.0546	0.0000	0.0249	0.0408	0.8450	0.0000	0.0347
6	0.1500	0.0000	0.0500	0.0500	0.0000	0.6500	0.1000
7	0.0000	0.0000	0.0000	0.0000	0.0000	0.0000	1.0000

Age 48	1	2	3	4	5	6	7
1	0.7896	0.0703	0.0669	0.0713	0.0007	0.0003	0.0009
2	0.5459	0.1858	0.1102	0.1542	0.0000	0.0039	0.0000
3	0.2862	0.0806	0.4608	0.1700	0.0000	0.0000	0.0024
4	0.1112	0.0260	0.0626	0.7869	0.0067	0.0035	0.0031
5	0.0561	0.0000	0.0273	0.0470	0.8305	0.0000	0.0391
6	0.1500	0.0000	0.0500	0.0500	0.0000	0.6500	0.1000
7	0.0000	0.0000	0.0000	0.0000	0.0000	0.0000	1.0000

Age 49	1	2	3	4	5	6	7
1	0.7854	0.0694	0.0679	0.0749	0.0009	0.0003	0.0012
2	0.5383	0.1830	0.1106	0.1638	0.0000	0.0043	0.0000
3	0.2757	0.0810	0.4655	0.1753	0.0000	0.0000	0.0024
4	0.1024	0.0237	0.0597	0.8005	0.0066	0.0038	0.0033
5	0.0570	0.0000	0.0289	0.0562	0.8151	0.0000	0.0428
6	0.1500	0.0000	0.0500	0.0500	0.0000	0.6500	0.1000
7	0.0000	0.0000	0.0000	0.0000	0.0000	0.0000	1.0000

Age 50	1	2	3	4	5	6	7
1	0.7813	0.0685	0.0689	0.0785	0.0010	0.0003	0.0015
2	0.5302	0.1800	0.1121	0.1731	0.0000	0.0046	0.0000
3	0.2666	0.0805	0.4705	0.1799	0.0001	0.0001	0.0024
4	0.0944	0.0216	0.0570	0.8127	0.0067	0.0040	0.0036
5	0.0571	0.0000	0.0294	0.0685	0.7993	0.0000	0.0457
6	0.1500	0.0000	0.0500	0.0500	0.0000	0.6500	0.1000
7	0.0000	0.0000	0.0000	0.0000	0.0000	0.0000	1.0000

Age 51	1	2	3	4	5	6	7
1	0.7770	0.0678	0.0698	0.0820	0.0012	0.0004	0.0018
2	0.5217	0.1769	0.1146	0.1818	0.0000	0.0049	0.0001
3	0.2588	0.0791	0.4755	0.1838	0.0001	0.0003	0.0023
4	0.0872	0.0197	0.0544	0.8236	0.0070	0.0041	0.0040
5	0.0564	0.0000	0.0288	0.0841	0.7824	0.0008	0.0475
6	0.1500	0.0000	0.0500	0.0500	0.0000	0.6500	0.1000
7	0.0000	0.0000	0.0000	0.0000	0.0000	0.0000	1.0000

Age 52	1	2	3	4	5	6	7
1	0.7727	0.0671	0.0707	0.0856	0.0014	0.0004	0.0021
2	0.5132	0.1739	0.1178	0.1898	0.0000	0.0051	0.0002
3	0.2521	0.0771	0.4807	0.1874	0.0001	0.0005	0.0021
4	0.0808	0.0180	0.0519	0.8335	0.0073	0.0041	0.0044
5	0.0550	0.0000	0.0271	0.1025	0.7651	0.0018	0.0485
6	0.1500	0.0000	0.0500	0.0500	0.0000	0.6500	0.1000
7	0.0000	0.0000	0.0000	0.0000	0.0000	0.0000	1.0000

Age 53	1	2	3	4	5	6	7
1	0.7682	0.0666	0.0715	0.0893	0.0015	0.0005	0.0024
2	0.5044	0.1713	0.1217	0.1969	0.0000	0.0053	0.0004
3	0.2461	0.0746	0.4858	0.1909	0.0001	0.0007	0.0019
4	0.0749	0.0165	0.0495	0.8423	0.0078	0.0041	0.0049
5	0.0530	0.0000	0.0246	0.1229	0.7476	0.0030	0.0489
6	0.1500	0.0000	0.0500	0.0500	0.0000	0.6500	0.1000
7	0.0000	0.0000	0.0000	0.0000	0.0000	0.0000	1.0000

Age 54	1	2	3	4	5	6	7
1	0.7634	0.0660	0.0724	0.0930	0.0018	0.0007	0.0027
2	0.4959	0.1691	0.1260	0.2029	0.0000	0.0054	0.0008
3	0.2405	0.0718	0.4906	0.1944	0.0001	0.0010	0.0017
4	0.0696	0.0151	0.0473	0.8503	0.0084	0.0040	0.0053
5	0.0505	0.0000	0.0214	0.1445	0.7303	0.0043	0.0490
6	0.1500	0.0000	0.0500	0.0500	0.0000	0.6500	0.1000
7	0.0000	0.0000	0.0000	0.0000	0.0000	0.0000	1.0000

Age 55	1	2	3	4	5	6	7
1	0.7586	0.0655	0.0732	0.0968	0.0020	0.0009	0.0030
2	0.4875	0.1677	0.1307	0.2076	0.0000	0.0054	0.0012
3	0.2350	0.0689	0.4952	0.1981	0.0001	0.0012	0.0016
4	0.0648	0.0139	0.0452	0.8572	0.0091	0.0040	0.0058
5	0.0475	0.0000	0.0178	0.1668	0.7131	0.0058	0.0490
6	0.0938	0.0000	0.0938	0.2812	0.0000	0.3749	0.1563
7	0.0000	0.0000	0.0000	0.0000	0.0000	0.0000	1.0000

Age 56	1	2	3	4	5	6	7
1	0.7535	0.0650	0.0741	0.1008	0.0022	0.0012	0.0032
2	0.4793	0.1672	0.1355	0.2109	0.0000	0.0053	0.0018
3	0.2294	0.0661	0.4992	0.2024	0.0001	0.0014	0.0014
4	0.0604	0.0127	0.0432	0.8635	0.0099	0.0040	0.0063
5	0.0441	0.0000	0.0139	0.1888	0.6966	0.0074	0.0492
6	0.0938	0.0000	0.0938	0.2812	0.0000	0.3749	0.1563
7	0.0000	0.0000	0.0000	0.0000	0.0000	0.0000	1.0000

2	0.4317	0.2010	0.2312	0.1361	0.0000	0.0000	0.0000
3	0.2017	0.0690	0.5904	0.1366	0.0007	0.0010	0.0006
4	0.0915	0.0308	0.1189	0.7505	0.0036	0.0014	0.0033
5	0.0000	0.0000	0.0199	0.1194	0.8604	0.0003	0.0000
6	0.0789	0.0587	0.0244	0.1575	0.0000	0.6522	0.0283
7	0.0000	0.0000	0.0000	0.0000	0.0000	0.0000	1.0000

Age 48	1	2	3	4	5	6	7
1	0.7630	0.0818	0.0998	0.0546	0.0003	0.0005	0.0000
2	0.4219	0.2017	0.2338	0.1426	0.0000	0.0000	0.0000
3	0.1930	0.0666	0.5954	0.1426	0.0007	0.0011	0.0006
4	0.0858	0.0309	0.1151	0.7596	0.0039	0.0014	0.0033
5	0.0000	0.0000	0.0221	0.1295	0.8482	0.0002	0.0000
6	0.0756	0.0473	0.0303	0.1500	0.0000	0.6641	0.0327
7	0.0000	0.0000	0.0000	0.0000	0.0000	0.0000	1.0000

Age 49	1	2	3	4	5	6	7
1	0.7614	0.0800	0.0992	0.0586	0.0004	0.0004	0.0000
2	0.4138	0.2026	0.2349	0.1487	0.0000	0.0000	0.0000
3	0.1847	0.0643	0.6001	0.1484	0.0006	0.0013	0.0006
4	0.0806	0.0306	0.1117	0.7680	0.0043	0.0016	0.0032
5	0.0000	0.0000	0.0238	0.1429	0.8333	0.0000	0.0000
6	0.0741	0.0370	0.0370	0.1481	0.0000	0.6668	0.0370
7	0.0000	0.0000	0.0000	0.0000	0.0000	0.0000	1.0000

Age 50	1	2	3	4	5	6	7
1	0.7593	0.0782	0.0984	0.0632	0.0004	0.0003	0.0002
2	0.4077	0.2035	0.2344	0.1542	0.0000	0.0000	0.0002
3	0.1771	0.0621	0.6043	0.1539	0.0006	0.0014	0.0006
4	0.0758	0.0298	0.1089	0.7758	0.0047	0.0019	0.0031
5	0.0030	0.0000	0.0249	0.1589	0.8119	0.0000	0.0013
6	0.0739	0.0284	0.0446	0.1529	0.0000	0.6589	0.0413
7	0.0000	0.0000	0.0000	0.0000	0.0000	0.0000	1.0000

Age 51	1	2	3	4	5	6	7
1	0.7568	0.0763	0.0975	0.0684	0.0004	0.0002	0.0004
2	0.4036	0.2044	0.2325	0.1591	0.0000	0.0000	0.0004
3	0.1702	0.0600	0.6080	0.1591	0.0005	0.0016	0.0006
4	0.0714	0.0285	0.1065	0.7833	0.0051	0.0023	0.0029
5	0.0067	0.0000	0.0255	0.1772	0.7875	0.0000	0.0031
6	0.0748	0.0213	0.0527	0.1634	0.0000	0.6422	0.0456
7	0.0000	0.0000	0.0000	0.0000	0.0000	0.0000	1.0000

Age 52	1	2	3	4	5	6	7
1	0.7539	0.0745	0.0965	0.0740	0.0004	0.0001	0.0006
2	0.4010	0.2053	0.2298	0.1633	0.0000	0.0000	0.0006
3	0.1638	0.0579	0.6115	0.1641	0.0005	0.0017	0.0005
4	0.0674	0.0268	0.1045	0.7903	0.0055	0.0028	0.0027
5	0.0108	0.0000	0.0255	0.1971	0.7614	0.0000	0.0052
6	0.0763	0.0156	0.0611	0.1783	0.0000	0.6189	0.0498
7	0.0000	0.0000	0.0000	0.0000	0.0000	0.0000	1.0000

Age 53	1	2	3	4	5	6	7
1	0.7507	0.0726	0.0956	0.0798	0.0004	0.0001	0.0008
2	0.3997	0.2059	0.2267	0.1669	0.0000	0.0000	0.0008
3	0.1578	0.0558	0.6149	0.1689	0.0004	0.0018	0.0004
4	0.0637	0.0247	0.1029	0.7970	0.0059	0.0033	0.0025
5	0.0151	0.0000	0.0252	0.2176	0.7343	0.0000	0.0078
6	0.0782	0.0110	0.0694	0.1962	0.0000	0.5912	0.0540
7	0.0000	0.0000	0.0000	0.0000	0.0000	0.0000	1.0000

Age 54	1	2	3	4	5	6	7
1	0.7472	0.0707	0.0948	0.0857	0.0004	0.0001	0.0011
2	0.3992	0.2062	0.2237	0.1699	0.0000	0.0000	0.0009
3	0.1521	0.0538	0.6177	0.1737	0.0004	0.0020	0.0003
4	0.0603	0.0225	0.1015	0.8033	0.0063	0.0038	0.0023
5	0.0195	0.0000	0.0244	0.2379	0.7073	0.0000	0.0109
6	0.0801	0.0075	0.0773	0.2159	0.0000	0.5610	0.0582
7	0.0000	0.0000	0.0000	0.0000	0.0000	0.0000	1.0000

Age 55	1	2	3	4	5	6	7
1	0.7439	0.0688	0.0941	0.0914	0.0004	0.0001	0.0013
2	0.3994	0.2062	0.2212	0.1722	0.0000	0.0000	0.0010
3	0.1466	0.0519	0.6205	0.1784	0.0003	0.0021	0.0002
4	0.0570	0.0201	0.1002	0.8096	0.0067	0.0043	0.0021
5	0.0236	0.0000	0.0234	0.2570	0.6815	0.0000	0.0145
6	0.0817	0.0048	0.0844	0.2360	0.0000	0.5308	0.0623
7	0.0000	0.0000	0.0000	0.0000	0.0000	0.0000	1.0000

Age 56	1	2	3	4	5	6	7
1	0.7407	0.0669	0.0937	0.0968	0.0003	0.0002	0.0014
2	0.3999	0.2056	0.2196	0.1738	0.0000	0.0000	0.0010
3	0.1412	0.0500	0.6231	0.1832	0.0002	0.0022	0.0001
4	0.0539	0.0177	0.0989	0.8157	0.0071	0.0047	0.0020
5	0.0273	0.0000	0.0221	0.2742	0.6579	0.0000	0.0185
6	0.0827	0.0029	0.0904	0.2553	0.0000	0.5023	0.0664
7	0.0000	0.0000	0.0000	0.0000	0.0000	0.0000	1.0000

Age 57	1	2	3	4	5	6	7
1	0.7482	0.0645	0.0751	0.1049	0.0025	0.0015	0.0033
2	0.4716	0.1677	0.1404	0.2125	0.0000	0.0052	0.0025
3	0.2233	0.0635	0.5028	0.2074	0.0000	0.0015	0.0014
4	0.0563	0.0117	0.0414	0.8690	0.0108	0.0041	0.0067
5	0.0404	0.0000	0.0100	0.2097	0.6810	0.0090	0.0499
6	0.0938	0.0000	0.0938	0.2812	0.0000	0.3749	0.1563
7	0.0000	0.0000	0.0000	0.0000	0.0000	0.0000	1.0000

Age 58	1	2	3	4	5	6	7
1	0.7426	0.0639	0.0761	0.1093	0.0028	0.0020	0.0033
2	0.4644	0.1696	0.1451	0.2124	0.0000	0.0050	0.0035
3	0.2165	0.0615	0.5056	0.2133	0.0000	0.0016	0.0015
4	0.0525	0.0108	0.0397	0.8738	0.0118	0.0043	0.0071
5	0.0365	0.0000	0.0062	0.2287	0.6666	0.0108	0.0512
6	0.0938	0.0000	0.0938	0.2812	0.0000	0.3749	0.1563
7	0.0000	0.0000	0.0000	0.0000	0.0000	0.0000	1.0000

Age 59	1	2	3	4	5	6	7
1	0.7368	0.0633	0.0772	0.1139	0.0032	0.0025	0.0032
2	0.4579	0.1729	0.1495	0.2103	0.0000	0.0047	0.0047
3	0.2087	0.0601	0.5074	0.2204	0.0000	0.0017	0.0017
4	0.0488	0.0100	0.0381	0.8782	0.0128	0.0046	0.0075
5	0.0325	0.0000	0.0028	0.2448	0.6538	0.0125	0.0536
6	0.0938	0.0000	0.0938	0.2812	0.0000	0.3749	0.1563
7	0.0000	0.0000	0.0000	0.0000	0.0000	0.0000	1.0000

Age 60	1	2	3	4	5	6	7
1	0.7309	0.0625	0.0783	0.1187	0.0035	0.0032	0.0029
2	0.4522	0.1778	0.1535	0.2061	0.0000	0.0043	0.0061
3	0.1996	0.0595	0.5086	0.2287	0.0000	0.0016	0.0020
4	0.0452	0.0092	0.0367	0.8821	0.0139	0.0051	0.0078
5	0.0286	0.0000	0.0000	0.2571	0.6429	0.0143	0.0571
6	0.0938	0.0000	0.0938	0.2812	0.0000	0.3749	0.1563
7	0.0000	0.0000	0.0000	0.0000	0.0000	0.0000	1.0000

Age 61	1	2	3	4	5	6	7
1	0.7245	0.0617	0.0796	0.1238	0.0039	0.0039	0.0026
2	0.4469	0.1841	0.1571	0.2004	0.0000	0.0038	0.0076
3	0.1896	0.0595	0.5089	0.2380	0.0000	0.0015	0.0025
4	0.0417	0.0085	0.0353	0.8856	0.0151	0.0057	0.0081
5	0.0247	0.0000	0.0000	0.2645	0.6328	0.0160	0.0620
6	0.0938	0.0000	0.0938	0.2812	0.0000	0.3749	0.1563
7	0.0000	0.0000	0.0000	0.0000	0.0000	0.0000	1.0000

Age 62	1	2	3	4	5	6	7
1	0.7183	0.0608	0.0808	0.1291	0.0042	0.0047	0.0021
2	0.4418	0.1913	0.1603	0.1939	0.0000	0.0033	0.0093
3	0.1790	0.0600	0.5086	0.2480	0.0000	0.0014	0.0030
4	0.0383	0.0078	0.0341	0.8888	0.0163	0.0064	0.0083
5	0.0209	0.0000	0.0000	0.2680	0.6255	0.0176	0.0680
6	0.0938	0.0000	0.0938	0.2812	0.0000	0.3749	0.1563
7	0.0000	0.0000	0.0000	0.0000	0.0000	0.0000	1.0000

Age 63	1	2	3	4	5	6	7
1	0.7120	0.0598	0.0820	0.1345	0.0045	0.0055	0.0017
2	0.4366	0.1993	0.1633	0.1871	0.0000	0.0028	0.0109
3	0.1682	0.0606	0.5081	0.2584	0.0000	0.0012	0.0035
4	0.0351	0.0073	0.0329	0.8913	0.0176	0.0073	0.0085
5	0.0174	0.0000	0.0000	0.2683	0.6202	0.0192	0.0749
6	0.0938	0.0000	0.0938	0.2812	0.0000	0.3749	0.1563
7	0.0000	0.0000	0.0000	0.0000	0.0000	0.0000	1.0000

Age 64	1	2	3	4	5	6	7
1	0.7059	0.0587	0.0831	0.1401	0.0048	0.0062	0.0012
2	0.4311	0.2075	0.1660	0.1807	0.0000	0.0023	0.0125
3	0.1576	0.0613	0.5074	0.2688	0.0000	0.0010	0.0039
4	0.0321	0.0067	0.0318	0.8935	0.0190	0.0082	0.0087
5	0.0141	0.0000	0.0000	0.2664	0.6164	0.0207	0.0824
6	0.0938	0.0000	0.0938	0.2812	0.0000	0.3749	0.1563
7	0.0000	0.0000	0.0000	0.0000	0.0000	0.0000	1.0000

Age 65	1	2	3	4	5	6	7
1	0.7002	0.0575	0.0840	0.1457	0.0049	0.0069	0.0008
2	0.4248	0.2158	0.1684	0.1754	0.0000	0.0017	0.0139
3	0.1475	0.0617	0.5068	0.2789	0.0000	0.0008	0.0043
4	0.0292	0.0062	0.0307	0.8953	0.0205	0.0091	0.0090
5	0.0110	0.0000	0.0000	0.2628	0.6141	0.0221	0.0900
6	0.0154	0.0000	0.0308	0.1538	0.0154	0.5846	0.2000
7	0.0000	0.0000	0.0000	0.0000	0.0000	0.0000	1.0000

Age 66	1	2	3	4	5	6	7
1	0.6951	0.0561	0.0847	0.1514	0.0049	0.0074	0.0004
2	0.4176	0.2237	0.1708	0.1717	0.0000	0.0012	0.0150
3	0.1385	0.0616	0.5064	0.2884	0.0000	0.0006	0.0045
4	0.0266	0.0057	0.0295	0.8967	0.0220	0.0102	0.0093

Age 57	1	2	3	4	5	6	7
1	0.7376	0.0650	0.0935	0.1018	0.0003	0.0003	0.0015
2	0.4004	0.2045	0.2196	0.1747	0.0000	0.0000	0.0008
3	0.1358	0.0482	0.6253	0.1880	0.0002	0.0024	0.0001
4	0.0509	0.0153	0.0976	0.8218	0.0074	0.0051	0.0019
5	0.0304	0.0000	0.0205	0.2885	0.6375	0.0000	0.0231
6	0.0827	0.0015	0.0949	0.2723	0.0000	0.4781	0.0705
7	0.0000	0.0000	0.0000	0.0000	0.0000	0.0000	1.0000

Age 58	1	2	3	4	5	6	7
1	0.7349	0.0631	0.0937	0.1062	0.0001	0.0005	0.0015
2	0.4005	0.2026	0.2214	0.1749	0.0000	0.0000	0.0005
3	0.1303	0.0464	0.6277	0.1930	0.0001	0.0025	0.0000
4	0.0479	0.0131	0.0962	0.8278	0.0077	0.0054	0.0019
5	0.0327	0.0000	0.0188	0.2991	0.6212	0.0000	0.0282
6	0.0814	0.0006	0.0976	0.2857	0.0000	0.4602	0.0745
7	0.0000	0.0000	0.0000	0.0000	0.0000	0.0000	1.0000

Age 59	1	2	3	4	5	6	7
1	0.7323	0.0613	0.0944	0.1099	0.0000	0.0007	0.0014
2	0.4000	0.2000	0.2255	0.1745	0.0000	0.0000	0.0000
3	0.1246	0.0447	0.6299	0.1982	0.0000	0.0026	0.0000
4	0.0449	0.0112	0.0946	0.8337	0.0080	0.0056	0.0020
5	0.0339	0.0000	0.0169	0.3051	0.6102	0.0000	0.0339
6	0.0784	0.0000	0.0980	0.2941	0.0000	0.4510	0.0784
7	0.0000	0.0000	0.0000	0.0000	0.0000	0.0000	1.0000

Age 60	1	2	3	4	5	6	7
1	0.7304	0.0594	0.0954	0.1126	0.0000	0.0010	0.0012
2	0.3983	0.1964	0.2321	0.1732	0.0000	0.0000	0.0000
3	0.1185	0.0431	0.6319	0.2037	0.0000	0.0028	0.0000
4	0.0418	0.0096	0.0927	0.8398	0.0083	0.0056	0.0022
5	0.0339	0.0000	0.0150	0.3056	0.6045	0.0009	0.0401
6	0.0736	0.0000	0.0960	0.2964	0.0000	0.4518	0.0823
7	0.0000	0.0000	0.0000	0.0000	0.0000	0.0000	1.0000

Age 61	1	2	3	4	5	6	7
1	0.7286	0.0576	0.0969	0.1145	0.0000	0.0014	0.0010
2	0.3957	0.1920	0.2407	0.1717	0.0000	0.0000	0.0000
3	0.1123	0.0416	0.6337	0.2094	0.0000	0.0029	0.0001
4	0.0387	0.0084	0.0905	0.8458	0.0085	0.0056	0.0025
5	0.0328	0.0000	0.0130	0.3015	0.6040	0.0020	0.0467
6	0.0672	0.0000	0.0917	0.2933	0.0000	0.4618	0.0860
7	0.0000	0.0000	0.0000	0.0000	0.0000	0.0000	1.0000

Age 62	1	2	3	4	5	6	7
1	0.7273	0.0559	0.0985	0.1158	0.0000	0.0018	0.0007
2	0.3924	0.1870	0.2504	0.1703	0.0000	0.0000	0.0000
3	0.1060	0.0401	0.6354	0.2153	0.0000	0.0030	0.0002
4	0.0356	0.0074	0.0880	0.8520	0.0088	0.0054	0.0028
5	0.0309	0.0000	0.0111	0.2937	0.6077	0.0032	0.0534
6	0.0595	0.0000	0.0857	0.2858	0.0000	0.4791	0.0898
7	0.0000	0.0000	0.0000	0.0000	0.0000	0.0000	1.0000

Age 63	1	2	3	4	5	6	7
1	0.7264	0.0543	0.1001	0.1165	0.0000	0.0022	0.0005
2	0.3885	0.1816	0.2604	0.1695	0.0000	0.0000	0.0000
3	0.0997	0.0386	0.6367	0.2214	0.0000	0.0032	0.0004
4	0.0325	0.0067	0.0853	0.8582	0.0091	0.0051	0.0031
5	0.0283	0.0000	0.0091	0.2831	0.6150	0.0045	0.0600
6	0.0509	0.0000	0.0784	0.2750	0.0000	0.5020	0.0937
7	0.0000	0.0000	0.0000	0.0000	0.0000	0.0000	1.0000

Age 64	1	2	3	4	5	6	7
1	0.7255	0.0529	0.1017	0.1169	0.0000	0.0026	0.0004
2	0.3844	0.1759	0.2700	0.1698	0.0000	0.0000	0.0000
3	0.0935	0.0371	0.6380	0.2276	0.0000	0.0032	0.0006
4	0.0295	0.0061	0.0823	0.8644	0.0094	0.0049	0.0034
5	0.0252	0.0000	0.0072	0.2705	0.6249	0.0058	0.0664
6	0.0418	0.0000	0.0702	0.2618	0.0000	0.5285	0.0977
7	0.0000	0.0000	0.0000	0.0000	0.0000	0.0000	1.0000

Age 65	1	2	3	4	5	6	7
1	0.7248	0.0517	0.1030	0.1170	0.0000	0.0030	0.0005
2	0.3801	0.1700	0.2783	0.1715	0.0000	0.0000	0.0000
3	0.0876	0.0356	0.6387	0.2339	0.0000	0.0033	0.0009
4	0.0267	0.0058	0.0791	0.8702	0.0099	0.0046	0.0037
5	0.0218	0.0000	0.0055	0.2570	0.6364	0.0070	0.0723
6	0.0324	0.0000	0.0615	0.2474	0.0000	0.5568	0.1019
7	0.0000	0.0000	0.0000	0.0000	0.0000	0.0000	1.0000

Age 66	1	2	3	4	5	6	7
1	0.7242	0.0507	0.1040	0.1170	0.0000	0.0033	0.0008
2	0.3758	0.1643	0.2847	0.1752	0.0000	0.0000	0.0000
3	0.0820	0.0340	0.6391	0.2403	0.0000	0.0033	0.0013
4	0.0242	0.0055	0.0757	0.8758	0.0105	0.0044	0.0039

5	0.0082	0.0000	0.0000	0.2585	0.6124	0.0234	0.0975
6	0.0154	0.0000	0.0308	0.1538	0.0154	0.5846	0.2000
7	0.0000	0.0000	0.0000	0.0000	0.0000	0.0000	1.0000

Age 67	1	2	3	4	5	6	7
1	0.6903	0.0547	0.0852	0.1571	0.0048	0.0078	0.0001
2	0.4091	0.2309	0.1730	0.1704	0.0000	0.0007	0.0158
3	0.1307	0.0609	0.5068	0.2968	0.0000	0.0003	0.0045
4	0.0242	0.0053	0.0284	0.8976	0.0237	0.0112	0.0096
5	0.0057	0.0000	0.0000	0.2540	0.6112	0.0246	0.1045
6	0.0154	0.0000	0.0308	0.1538	0.0154	0.5846	0.2000
7	0.0000	0.0000	0.0000	0.0000	0.0000	0.0000	1.0000

Age 68	1	2	3	4	5	6	7
1	0.6862	0.0532	0.0854	0.1627	0.0045	0.0080	0.0000
2	0.3990	0.2371	0.1752	0.1721	0.0000	0.0003	0.0162
3	0.1247	0.0593	0.5076	0.3039	0.0000	0.0002	0.0043
4	0.0221	0.0049	0.0271	0.8983	0.0254	0.0122	0.0100
5	0.0035	0.0000	0.0000	0.2502	0.6100	0.0257	0.1106
6	0.0154	0.0000	0.0308	0.1538	0.0154	0.5846	0.2000
7	0.0000	0.0000	0.0000	0.0000	0.0000	0.0000	1.0000

Age 69	1	2	3	4	5	6	7
1	0.6832	0.0515	0.0851	0.1683	0.0040	0.0079	0.0000
2	0.3871	0.2419	0.1774	0.1774	0.0000	0.0000	0.0161
3	0.1208	0.0566	0.5094	0.3094	0.0000	0.0000	0.0038
4	0.0203	0.0045	0.0258	0.8984	0.0272	0.0133	0.0105
5	0.0016	0.0000	0.0000	0.2479	0.6081	0.0267	0.1157
6	0.0154	0.0000	0.0308	0.1538	0.0154	0.5846	0.2000
7	0.0000	0.0000	0.0000	0.0000	0.0000	0.0000	1.0000

Age 70	1	2	3	4	5	6	7
1	0.6810	0.0497	0.0845	0.1738	0.0032	0.0076	0.0002
2	0.3730	0.2451	0.1797	0.1867	0.0000	0.0000	0.0155
3	0.1192	0.0527	0.5122	0.3130	0.0000	0.0000	0.0029
4	0.0187	0.0042	0.0244	0.8981	0.0292	0.0143	0.0111
5	0.0000	0.0000	0.0000	0.2477	0.6055	0.0275	0.1193
6	0.0154	0.0000	0.0308	0.1538	0.0154	0.5846	0.2000
7	0.0000	0.0000	0.0000	0.0000	0.0000	0.0000	1.0000

Age 71	1	2	3	4	5	6	7
1	0.6795	0.0478	0.0835	0.1792	0.0024	0.0070	0.0006
2	0.3573	0.2467	0.1821	0.1995	0.0000	0.0000	0.0144
3	0.1196	0.0476	0.5158	0.3151	0.0000	0.0000	0.0019
4	0.0174	0.0039	0.0230	0.8975	0.0312	0.0152	0.0118
5	0.0000	0.0000	0.0005	0.2498	0.6005	0.0282	0.1210
6	0.0154	0.0000	0.0308	0.1538	0.0154	0.5846	0.2000
7	0.0000	0.0000	0.0000	0.0000	0.0000	0.0000	1.0000

Age 72	1	2	3	4	5	6	7
1	0.6785	0.0457	0.0822	0.1845	0.0015	0.0063	0.0013
2	0.3403	0.2470	0.1848	0.2150	0.0000	0.0000	0.0129
3	0.1218	0.0418	0.5194	0.3163	0.0000	0.0000	0.0007
4	0.0164	0.0036	0.0214	0.8964	0.0332	0.0161	0.0129
5	0.0000	0.0000	0.0009	0.2543	0.5947	0.0287	0.1214
6	0.0154	0.0000	0.0308	0.1538	0.0154	0.5846	0.2000
7	0.0000	0.0000	0.0000	0.0000	0.0000	0.0000	1.0000

Age 73	1	2	3	4	5	6	7
1	0.6780	0.0436	0.0805	0.1898	0.0005	0.0054	0.0022
2	0.3225	0.2460	0.1880	0.2324	0.0000	0.0000	0.0111
3	0.1252	0.0354	0.5227	0.3167	0.0000	0.0000	0.0000
4	0.0156	0.0033	0.0199	0.8945	0.0353	0.0170	0.0144
5	0.0000	0.0000	0.0011	0.2606	0.5883	0.0290	0.1210
6	0.0154	0.0000	0.0308	0.1538	0.0154	0.5846	0.2000
7	0.0000	0.0000	0.0000	0.0000	0.0000	0.0000	1.0000

Age 74	1	2	3	4	5	6	7
1	0.6775	0.0413	0.0784	0.1949	0.0000	0.0045	0.0034
2	0.3043	0.2439	0.1917	0.2510	0.0000	0.0000	0.0091
3	0.1294	0.0287	0.5249	0.3170	0.0000	0.0000	0.0000
4	0.0150	0.0030	0.0184	0.8919	0.0375	0.0178	0.0164
5	0.0000	0.0000	0.0011	0.2681	0.5813	0.0291	0.1204
6	0.0154	0.0000	0.0308	0.1538	0.0154	0.5846	0.2000
7	0.0000	0.0000	0.0000	0.0000	0.0000	0.0000	1.0000

Age 75	1	2	3	4	5	6	7
1	0.6769	0.0389	0.0760	0.1998	0.0000	0.0035	0.0049
2	0.2861	0.2409	0.1960	0.2699	0.0000	0.0000	0.0071
3	0.1340	0.0220	0.5261	0.3179	0.0000	0.0000	0.0000
4	0.0145	0.0028	0.0169	0.8888	0.0396	0.0185	0.0189
5	0.0000	0.0000	0.0010	0.2761	0.5739	0.0291	0.1199
6	0.0133	0.0000	0.0000	0.1733	0.0133	0.5468	0.2533
7	0.0000	0.0000	0.0000	0.0000	0.0000	0.0000	1.0000

Age 76	1	2	3	4	5	6	7
1	0.6763	0.0364	0.0733	0.2048	0.0000	0.0025	0.0067

5	0.0184	0.0000	0.0038	0.2435	0.6489	0.0080	0.0774
6	0.0232	0.0000	0.0527	0.2327	0.0000	0.5852	0.1062
7	0.0000	0.0000	0.0000	0.0000	0.0000	0.0000	1.0000

Age 67	1	2	3	4	5	6	7
1	0.7237	0.0500	0.1044	0.1170	0.0000	0.0035	0.0014
2	0.3717	0.1587	0.2882	0.1814	0.0000	0.0000	0.0000
3	0.0769	0.0324	0.6389	0.2467	0.0000	0.0033	0.0018
4	0.0218	0.0053	0.0721	0.8813	0.0112	0.0042	0.0041
5	0.0151	0.0000	0.0023	0.2307	0.6613	0.0089	0.0817
6	0.0145	0.0000	0.0443	0.2187	0.0000	0.6116	0.1109
7	0.0000	0.0000	0.0000	0.0000	0.0000	0.0000	1.0000

Age 68	1	2	3	4	5	6	7
1	0.7230	0.0497	0.1041	0.1172	0.0000	0.0037	0.0023
2	0.3680	0.1534	0.2882	0.1904	0.0000	0.0000	0.0000
3	0.0725	0.0308	0.6379	0.2532	0.0000	0.0032	0.0024
4	0.0198	0.0051	0.0684	0.8864	0.0120	0.0042	0.0041
5	0.0121	0.0000	0.0010	0.2198	0.6729	0.0094	0.0848
6	0.0067	0.0000	0.0367	0.2064	0.0000	0.6343	0.1159
7	0.0000	0.0000	0.0000	0.0000	0.0000	0.0000	1.0000

Age 69	1	2	3	4	5	6	7
1	0.7225	0.0496	0.1029	0.1176	0.0000	0.0037	0.0037
2	0.3649	0.1486	0.2838	0.2027	0.0000	0.0000	0.0000
3	0.0687	0.0290	0.6366	0.2595	0.0000	0.0031	0.0031
4	0.0181	0.0050	0.0645	0.8911	0.0131	0.0043	0.0039
5	0.0096	0.0000	0.0000	0.2115	0.6828	0.0096	0.0865
6	0.0000	0.0000	0.0303	0.1970	0.0000	0.6515	0.1212
7	0.0000	0.0000	0.0000	0.0000	0.0000	0.0000	1.0000

Age 70	1	2	3	4	5	6	7
1	0.7211	0.0500	0.1008	0.1185	0.0006	0.0036	0.0055
2	0.3614	0.1441	0.2737	0.2182	0.0000	0.0000	0.0027
3	0.0657	0.0272	0.6345	0.2658	0.0002	0.0028	0.0038
4	0.0168	0.0048	0.0605	0.8954	0.0144	0.0045	0.0036
5	0.0078	0.0000	0.0000	0.2065	0.6895	0.0094	0.0868
6	0.0000	0.0001	0.0253	0.1900	0.0000	0.6583	0.1263
7	0.0000	0.0000	0.0000	0.0000	0.0000	0.0000	1.0000

Age 71	1	2	3	4	5	6	7
1	0.7195	0.0506	0.0979	0.1198	0.0012	0.0033	0.0077
2	0.3581	0.1399	0.2592	0.2367	0.0000	0.0000	0.0061
3	0.0634	0.0252	0.6316	0.2719	0.0005	0.0026	0.0048
4	0.0158	0.0045	0.0564	0.8993	0.0159	0.0048	0.0033
5	0.0066	0.0000	0.0000	0.2045	0.6941	0.0089	0.0859
6	0.0000	0.0002	0.0217	0.1864	0.0000	0.6598	0.1319
7	0.0000	0.0000	0.0000	0.0000	0.0000	0.0000	1.0000

Age 72	1	2	3	4	5	6	7
1	0.7177	0.0514	0.0942	0.1215	0.0020	0.0030	0.0102
2	0.3546	0.1359	0.2412	0.2579	0.0000	0.0000	0.0104
3	0.0617	0.0232	0.6285	0.2777	0.0008	0.0023	0.0058
4	0.0152	0.0042	0.0523	0.9024	0.0176	0.0052	0.0031
5	0.0060	0.0000	0.0000	0.2051	0.6966	0.0080	0.0843
6	0.0000	0.0002	0.0194	0.1856	0.0000	0.6570	0.1378
7	0.0000	0.0000	0.0000	0.0000	0.0000	0.0000	1.0000

Age 73	1	2	3	4	5	6	7
1	0.7155	0.0523	0.0900	0.1236	0.0028	0.0026	0.0132
2	0.3506	0.1320	0.2207	0.2811	0.0000	0.0000	0.0156
3	0.0604	0.0212	0.6250	0.2832	0.0012	0.0019	0.0071
4	0.0148	0.0039	0.0482	0.9048	0.0194	0.0057	0.0032
5	0.0058	0.0000	0.0000	0.2076	0.6971	0.0070	0.0825
6	0.0000	0.0002	0.0180	0.1868	0.0000	0.6510	0.1440
7	0.0000	0.0000	0.0000	0.0000	0.0000	0.0000	1.0000

Age 74	1	2	3	4	5	6	7
1	0.7131	0.0532	0.0853	0.1262	0.0036	0.0021	0.0165
2	0.3459	0.1280	0.1987	0.3058	0.0000	0.0000	0.0217
3	0.0594	0.0192	0.6214	0.2882	0.0017	0.0015	0.0086
4	0.0146	0.0036	0.0442	0.9061	0.0215	0.0062	0.0038
5	0.0059	0.0000	0.0000	0.2115	0.6960	0.0058	0.0808
6	0.0000	0.0002	0.0174	0.1894	0.0000	0.6426	0.1504
7	0.0000	0.0000	0.0000	0.0000	0.0000	0.0000	1.0000

Age 75	1	2	3	4	5	6	7
1	0.7102	0.0541	0.0804	0.1293	0.0043	0.0017	0.0200
2	0.3401	0.1236	0.1761	0.3314	0.0000	0.0000	0.0287
3	0.0586	0.0172	0.6178	0.2927	0.0022	0.0012	0.0103
4	0.0146	0.0033	0.0403	0.9063	0.0237	0.0067	0.0051
5	0.0063	0.0000	0.0000	0.2162	0.6933	0.0045	0.0797
6	0.0000	0.0002	0.0173	0.1927	0.0000	0.6329	0.1569
7	0.0000	0.0000	0.0000	0.0000	0.0000	0.0000	1.0000

Age 76	1	2	3	4	5	6	7
1	0.7069	0.0548	0.0754	0.1328	0.0050	0.0012	0.0239

2	0.2684	0.2371	0.2011	0.2884	0.0000	0.0000	0.0050
3	0.1388	0.0156	0.5258	0.3198	0.0000	0.0000	0.0000
4	0.0142	0.0026	0.0155	0.8847	0.0417	0.0191	0.0222
5	0.0000	0.0000	0.0009	0.2841	0.5663	0.0288	0.1199
6	0.0133	0.0000	0.0000	0.1733	0.0133	0.5468	0.2533
7	0.0000	0.0000	0.0000	0.0000	0.0000	0.0000	1.0000

Age 77	1	2	3	4	5	6	7
1	0.6759	0.0338	0.0703	0.2097	0.0000	0.0015	0.0088
2	0.2515	0.2326	0.2071	0.3056	0.0000	0.0000	0.0031
3	0.1433	0.0096	0.5237	0.3234	0.0000	0.0000	0.0000
4	0.0139	0.0025	0.0142	0.8797	0.0438	0.0197	0.0262
5	0.0000	0.0000	0.0007	0.2913	0.5587	0.0282	0.1211
6	0.0133	0.0000	0.0000	0.1733	0.0133	0.5468	0.2533
7	0.0000	0.0000	0.0000	0.0000	0.0000	0.0000	1.0000

Age 78	1	2	3	4	5	6	7
1	0.6749	0.0311	0.0672	0.2148	0.0000	0.0007	0.0113
2	0.2360	0.2276	0.2141	0.3209	0.0000	0.0000	0.0014
3	0.1472	0.0043	0.5194	0.3291	0.0000	0.0000	0.0000
4	0.0138	0.0024	0.0130	0.8737	0.0459	0.0201	0.0311
5	0.0000	0.0000	0.0004	0.2972	0.5513	0.0273	0.1238
6	0.0133	0.0000	0.0000	0.1733	0.0133	0.5468	0.2533
7	0.0000	0.0000	0.0000	0.0000	0.0000	0.0000	1.0000

Age 79	1	2	3	4	5	6	7
1	0.6737	0.0284	0.0638	0.2199	0.0000	0.0000	0.0142
2	0.2222	0.2222	0.2222	0.3333	0.0000	0.0000	0.0000
3	0.1500	0.0000	0.5125	0.3375	0.0000	0.0000	0.0000
4	0.0137	0.0023	0.0120	0.8666	0.0479	0.0205	0.0370
5	0.0000	0.0000	0.0002	0.3012	0.5438	0.0262	0.1286
6	0.0133	0.0000	0.0000	0.1733	0.0133	0.5468	0.2533
7	0.0000	0.0000	0.0000	0.0000	0.0000	0.0000	1.0000

Age 80	1	2	3	4	5	6	7
1	0.6708	0.0255	0.0602	0.2246	0.0015	0.0000	0.0174
2	0.2103	0.2164	0.2312	0.3420	0.0000	0.0001	0.0000
3	0.1507	0.0000	0.4998	0.3471	0.0000	0.0000	0.0024
4	0.0136	0.0022	0.0111	0.8585	0.0498	0.0208	0.0440
5	0.0000	0.0000	0.0000	0.3025	0.5370	0.0247	0.1358
6	0.0133	0.0000	0.0000	0.1733	0.0133	0.5468	0.2533
7	0.0000	0.0000	0.0000	0.0000	0.0000	0.0000	1.0000

Age 81	1	2	3	4	5	6	7
1	0.6673	0.0225	0.0563	0.2294	0.0034	0.0000	0.0211
2	0.2004	0.2104	0.2414	0.3476	0.0000	0.0001	0.0000
3	0.1501	0.0000	0.4847	0.3596	0.0000	0.0001	0.0056
4	0.0135	0.0023	0.0105	0.8493	0.0516	0.0209	0.0519
5	0.0012	0.0000	0.0000	0.3003	0.5300	0.0228	0.1457
6	0.0133	0.0000	0.0000	0.1733	0.0133	0.5468	0.2533
7	0.0000	0.0000	0.0000	0.0000	0.0000	0.0000	1.0000

Age 82	1	2	3	4	5	6	7
1	0.6633	0.0194	0.0523	0.2342	0.0058	0.0000	0.0250
2	0.1923	0.2044	0.2526	0.3505	0.0000	0.0001	0.0000
3	0.1485	0.0000	0.4673	0.3747	0.0000	0.0001	0.0094
4	0.0134	0.0023	0.0099	0.8394	0.0533	0.0210	0.0607
5	0.0025	0.0000	0.0000	0.2952	0.5234	0.0207	0.1582
6	0.0133	0.0000	0.0000	0.1733	0.0133	0.5468	0.2533
7	0.0000	0.0000	0.0000	0.0000	0.0000	0.0000	1.0000

Age 83	1	2	3	4	5	6	7
1	0.6586	0.0163	0.0482	0.2390	0.0087	0.0000	0.0292
2	0.1858	0.1982	0.2647	0.3512	0.0000	0.0001	0.0000
3	0.1460	0.0000	0.4481	0.3920	0.0000	0.0001	0.0139
4	0.0134	0.0024	0.0096	0.8283	0.0550	0.0211	0.0702
5	0.0039	0.0000	0.0000	0.2875	0.5174	0.0182	0.1730
6	0.0133	0.0000	0.0000	0.1733	0.0133	0.5468	0.2533
7	0.0000	0.0000	0.0000	0.0000	0.0000	0.0000	1.0000

Age 84	1	2	3	4	5	6	7
1	0.6537	0.0131	0.0440	0.2438	0.0118	0.0000	0.0336
2	0.1804	0.1920	0.2775	0.3499	0.0000	0.0001	0.0000
3	0.1427	0.0000	0.4272	0.4112	0.0000	0.0001	0.0188
4	0.0133	0.0024	0.0093	0.8169	0.0567	0.0210	0.0804
5	0.0054	0.0000	0.0000	0.2777	0.5116	0.0155	0.1898
6	0.0133	0.0000	0.0000	0.1733	0.0133	0.5468	0.2533
7	0.0000	0.0000	0.0000	0.0000	0.0000	0.0000	1.0000

Age 85	1	2	3	4	5	6	7
1	0.6486	0.0098	0.0396	0.2486	0.0152	0.0000	0.0382
2	0.1761	0.1858	0.2909	0.3471	0.0000	0.0001	0.0000
3	0.1389	0.0000	0.4050	0.4320	0.0000	0.0000	0.0241
4	0.0133	0.0025	0.0091	0.8049	0.0583	0.0209	0.0910
5	0.0070	0.0000	0.0000	0.2660	0.5061	0.0127	0.2082
6	0.0000	0.0000	0.0000	0.0000	0.0000	0.0000	0.0000
7	0.0000	0.0000	0.0000	0.0000	0.0000	0.0000	1.0000

2	0.3329	0.1188	0.1540	0.3576	0.0000	0.0000	0.0367
3	0.0579	0.0152	0.6146	0.2965	0.0028	0.0008	0.0122
4	0.0148	0.0030	0.0366	0.9053	0.0260	0.0071	0.0072
5	0.0070	0.0000	0.0000	0.2212	0.6891	0.0032	0.0795
6	0.0000	0.0001	0.0176	0.1960	0.0000	0.6229	0.1634
7	0.0000	0.0000	0.0000	0.0000	0.0000	0.0000	1.0000

Age 77	1	2	3	4	5	6	7
1	0.7034	0.0552	0.0704	0.1369	0.0055	0.0007	0.0279
2	0.3240	0.1134	0.1333	0.3836	0.0000	0.0000	0.0456
3	0.0571	0.0134	0.6113	0.2997	0.0035	0.0005	0.0145
4	0.0150	0.0027	0.0331	0.9031	0.0285	0.0074	0.0102
5	0.0076	0.0000	0.0000	0.2259	0.6837	0.0020	0.0808
6	0.0000	0.0001	0.0180	0.1988	0.0000	0.6133	0.1698
7	0.0000	0.0000	0.0000	0.0000	0.0000	0.0000	1.0000

Age 78	1	2	3	4	5	6	7
1	0.6994	0.0553	0.0655	0.1414	0.0059	0.0003	0.0322
2	0.3132	0.1072	0.1150	0.4090	0.0000	0.0000	0.0556
3	0.0561	0.0116	0.6089	0.3020	0.0042	0.0002	0.0170
4	0.0153	0.0024	0.0299	0.8993	0.0311	0.0076	0.0144
5	0.0083	0.0000	0.0000	0.2297	0.6772	0.0009	0.0839
6	0.0000	0.0000	0.0182	0.2004	0.0000	0.6055	0.1759
7	0.0000	0.0000	0.0000	0.0000	0.0000	0.0000	1.0000

Age 79	1	2	3	4	5	6	7
1	0.6951	0.0549	0.0610	0.1463	0.0061	0.0000	0.0366
2	0.3000	0.1000	0.1000	0.4333	0.0000	0.0000	0.0667
3	0.0547	0.0100	0.6069	0.3035	0.0050	0.0000	0.0199
4	0.0156	0.0021	0.0270	0.8940	0.0338	0.0076	0.0199
5	0.0089	0.0000	0.0000	0.2321	0.6697	0.0000	0.0893
6	0.0000	0.0000	0.0182	0.2000	0.0000	0.6000	0.1818
7	0.0000	0.0000	0.0000	0.0000	0.0000	0.0000	1.0000

Age 80	1	2	3	4	5	6	7
1	0.6902	0.0540	0.0569	0.1518	0.0060	0.0000	0.0411
2	0.2843	0.0917	0.0891	0.4561	0.0000	0.0000	0.0788
3	0.0530	0.0085	0.6057	0.3039	0.0058	0.0000	0.0231
4	0.0159	0.0019	0.0246	0.8868	0.0366	0.0074	0.0268
5	0.0094	0.0000	0.0005	0.2324	0.6606	0.0000	0.0971
6	0.0045	0.0000	0.0176	0.1963	0.0000	0.5952	0.1864
7	0.0000	0.0000	0.0000	0.0000	0.0000	0.0000	1.0000

Age 81	1	2	3	4	5	6	7
1	0.6850	0.0526	0.0532	0.1577	0.0058	0.0000	0.0458
2	0.2664	0.0825	0.0821	0.4773	0.0000	0.0000	0.0918
3	0.0508	0.0071	0.6051	0.3035	0.0068	0.0000	0.0267
4	0.0162	0.0017	0.0225	0.8780	0.0395	0.0071	0.0350
5	0.0096	0.0000	0.0009	0.2309	0.6513	0.0000	0.1073
6	0.0095	0.0000	0.0164	0.1902	0.0000	0.5933	0.1906
7	0.0000	0.0000	0.0000	0.0000	0.0000	0.0000	1.0000

Age 82	1	2	3	4	5	6	7
1	0.6796	0.0508	0.0498	0.1640	0.0053	0.0000	0.0505
2	0.2463	0.0723	0.0784	0.4973	0.0000	0.0000	0.1057
3	0.0483	0.0059	0.6052	0.3024	0.0077	0.0000	0.0305
4	0.0164	0.0015	0.0206	0.8681	0.0425	0.0066	0.0443
5	0.0097	0.0000	0.0014	0.2280	0.6413	0.0000	0.1196
6	0.0149	0.0000	0.0149	0.1819	0.0000	0.5941	0.1942
7	0.0000	0.0000	0.0000	0.0000	0.0000	0.0000	1.0000

Age 83	1	2	3	4	5	6	7
1	0.6739	0.0486	0.0468	0.1706	0.0047	0.0000	0.0554
2	0.2246	0.0614	0.0776	0.5161	0.0000	0.0000	0.1203
3	0.0454	0.0047	0.6059	0.3006	0.0088	0.0000	0.0346
4	0.0166	0.0013	0.0191	0.8570	0.0455	0.0059	0.0546
5	0.0097	0.0000	0.0019	0.2237	0.6310	0.0000	0.1337
6	0.0207	0.0000	0.0130	0.1719	0.0000	0.5969	0.1975
7	0.0000	0.0000	0.0000	0.0000	0.0000	0.0000	1.0000

Age 84	1	2	3	4	5	6	7
1	0.6682	0.0461	0.0440	0.1775	0.0039	0.0000	0.0603
2	0.2014	0.0499	0.0792	0.5340	0.0000	0.0000	0.1355
3	0.0424	0.0037	0.6070	0.2983	0.0098	0.0000	0.0388
4	0.0167	0.0012	0.0177	0.8448	0.0486	0.0052	0.0658
5	0.0096	0.0000	0.0024	0.2184	0.6203	0.0000	0.1493
6	0.0267	0.0000	0.0107	0.1604	0.0000	0.6017	0.2005
7	0.0000	0.0000	0.0000	0.0000	0.0000	0.0000	1.0000

Age 85	1	2	3	4	5	6	7
1	0.6622	0.0434	0.0415	0.1846	0.0030	0.0000	0.0653
2	0.1771	0.0379	0.0827	0.5511	0.0000	0.0000	0.1512
3	0.0391	0.0027	0.6085	0.2955	0.0109	0.0000	0.0433
4	0.0169	0.0011	0.0166	0.8318	0.0517	0.0043	0.0776
5	0.0095	0.0000	0.0029	0.2122	0.6093	0.0000	0.1661
6	0.0330	0.0000	0.0083	0.1476	0.0000	0.6079	0.2032
7	0.0000	0.0000	0.0000	0.0000	0.0000	0.0000	1.0000

Age 86	1	2	3	4	5	6	7
1	0.6430	0.0066	0.0352	0.2535	0.0188	0.0000	0.0429
2	0.1725	0.1794	0.3048	0.3432	0.0000	0.0001	0.0000
3	0.1345	0.0000	0.3817	0.4539	0.0000	0.0000	0.0298
4	0.0132	0.0027	0.0089	0.7926	0.0599	0.0208	0.1019
5	0.0086	0.0000	0.0000	0.2529	0.5010	0.0096	0.2279
6	0.0000	0.0000	0.0000	0.0000	0.0000	0.0000	0.0000
7	0.0000	0.0000	0.0000	0.0000	0.0000	0.0000	1.0000

Age 87	1	2	3	4	5	6	7
1	0.6373	0.0033	0.0308	0.2583	0.0225	0.0000	0.0478
2	0.1695	0.1731	0.3190	0.3384	0.0000	0.0000	0.0000
3	0.1298	0.0000	0.3578	0.4767	0.0000	0.0000	0.0357
4	0.0132	0.0028	0.0089	0.7799	0.0614	0.0207	0.1131
5	0.0104	0.0000	0.0000	0.2386	0.4959	0.0065	0.2486
6	0.0000	0.0000	0.0000	0.0000	0.0000	0.0000	0.0000
7	0.0000	0.0000	0.0000	0.0000	0.0000	0.0000	1.0000

Age 88	1	2	3	4	5	6	7
1	0.6316	0.0000	0.0263	0.2632	0.0263	0.0000	0.0526
2	0.1667	0.1667	0.3333	0.3333	0.0000	0.0000	0.0000
3	0.1250	0.0000	0.3333	0.5000	0.0000	0.0000	0.0417
4	0.0132	0.0029	0.0088	0.7671	0.0630	0.0205	0.1245
5	0.0121	0.0000	0.0000	0.2237	0.4910	0.0033	0.2700
6	0.0000	0.0000	0.0000	0.0000	0.0000	0.0000	0.0000
7	0.0000	0.0000	0.0000	0.0000	0.0000	0.0000	1.0000

Age 89	1	2	3	4	5	6	7
1	0.6236	0.0000	0.0218	0.2670	0.0300	0.0003	0.0573
2	0.1637	0.1602	0.3475	0.3280	0.0000	0.0000	0.0006
3	0.1199	0.0018	0.3083	0.5224	0.0000	0.0000	0.0476
4	0.0131	0.0031	0.0087	0.7545	0.0645	0.0203	0.1358
5	0.0139	0.0000	0.0000	0.2083	0.4861	0.0000	0.2917
6	0.0000	0.0000	0.0000	0.0000	0.0000	0.0000	0.0000
7	0.0000	0.0000	0.0000	0.0000	0.0000	0.0000	1.0000

Age >=90	1	2	3	4	5	6	7
1	0.6157	0.0000	0.0173	0.2709	0.0336	0.0006	0.0619
2	0.1606	0.1537	0.3615	0.3231	0.0000	0.0000	0.0012
3	0.1150	0.0035	0.2836	0.5445	0.0000	0.0000	0.0534
4	0.0131	0.0032	0.0086	0.7418	0.0661	0.0202	0.1470
5	0.0156	0.0000	0.0001	0.1924	0.4796	0.0000	0.3123
6	0.0000	0.0000	0.0000	0.0000	0.0000	0.0000	0.0000
7	0.0000	0.0000	0.0000	0.0000	0.0000	0.0000	1.0000

Age 86	1	2	3	4	5	6	7
1	0.6561	0.0405	0.0390	0.1920	0.0021	0.0000	0.0703
2	0.1519	0.0254	0.0877	0.5677	0.0000	0.0000	0.1673
3	0.0357	0.0018	0.6103	0.2924	0.0120	0.0000	0.0478
4	0.0170	0.0009	0.0155	0.8184	0.0549	0.0034	0.0899
5	0.0092	0.0000	0.0034	0.2053	0.5983	0.0000	0.1838
6	0.0395	0.0000	0.0056	0.1340	0.0000	0.6151	0.2058
7	0.0000	0.0000	0.0000	0.0000	0.0000	0.0000	1.0000

Age 87	1	2	3	4	5	6	7
1	0.6500	0.0375	0.0367	0.1994	0.0010	0.0000	0.0754
2	0.1261	0.0128	0.0936	0.5839	0.0000	0.0000	0.1836
3	0.0321	0.0009	0.6123	0.2891	0.0131	0.0000	0.0525
4	0.0172	0.0008	0.0146	0.8045	0.0580	0.0024	0.1025
5	0.0090	0.0000	0.0038	0.1980	0.5871	0.0000	0.2021
6	0.0460	0.0000	0.0028	0.1198	0.0000	0.6232	0.2082
7	0.0000	0.0000	0.0000	0.0000	0.0000	0.0000	1.0000

Age 88	1	2	3	4	5	6	7
1	0.6436	0.0345	0.0345	0.2069	0.0000	0.0000	0.0805
2	0.1000	0.0000	0.1000	0.6000	0.0000	0.0000	0.2000
3	0.0286	0.0000	0.6143	0.2857	0.0143	0.0000	0.0571
4	0.0173	0.0007	0.0137	0.7904	0.0612	0.0014	0.1153
5	0.0087	0.0000	0.0043	0.1905	0.5757	0.0000	0.2208
6	0.0526	0.0000	0.0000	0.1053	0.0000	0.6316	0.2105
7	0.0000	0.0000	0.0000	0.0000	0.0000	0.0000	1.0000

Age 89	1	2	3	4	5	6	7
1	0.6368	0.0314	0.0322	0.2141	0.0000	0.0001	0.0854
2	0.0730	0.0000	0.1051	0.6082	0.0000	0.0000	0.2137
3	0.0250	0.0000	0.6157	0.2820	0.0154	0.0001	0.0618
4	0.0174	0.0006	0.0128	0.7762	0.0644	0.0005	0.1281
5	0.0084	0.0000	0.0048	0.1829	0.5641	0.0004	0.2394
6	0.0591	0.0000	0.0000	0.0905	0.0000	0.6381	0.2123
7	0.0000	0.0000	0.0000	0.0000	0.0000	0.0000	1.0000

Age >=90	1	2	3	4	5	6	7
1	0.6299	0.0283	0.0298	0.2213	0.0000	0.0003	0.0904
2	0.0469	0.0000	0.1096	0.6166	0.0000	0.0000	0.2269
3	0.0214	0.0000	0.6171	0.2785	0.0165	0.0002	0.0663
4	0.0175	0.0005	0.0118	0.7620	0.0676	0.0000	0.1406
5	0.0081	0.0000	0.0053	0.1755	0.5528	0.0007	0.2576
6	0.0654	0.0000	0.0000	0.0761	0.0000	0.6444	0.2141
7	0.0000	0.0000	0.0000	0.0000	0.0000	0.0000	1.0000
